# Genomic Landscape of Endometrial, Ovarian, and Cervical Cancers in Japan from the Database in the Center for Cancer Genomics and Advanced Therapeutics

**DOI:** 10.3390/cancers16010136

**Published:** 2023-12-27

**Authors:** Qian Xi, Hidenori Kage, Miho Ogawa, Asami Matsunaga, Akira Nishijima, Kenbun Sone, Kei Kawana, Katsutoshi Oda

**Affiliations:** 1 Division of Integrative Genomics, Graduate School of Medicine, The University of Tokyo, Tokyo 113-8654, Japan; xiqianvictory@g.ecc.u-tokyo.ac.jp (Q.X.);; 2 Department of Respiratory Medicine, Graduate School of Medicine, The University of Tokyo, Tokyo 113-8654, Japan; 3 Next-Generation Precision Medicine Development Laboratory, Graduate School of Medicine, The University of Tokyo, Tokyo 113-8654, Japan; 4 Department of Obstetrics and Gynecology, Graduate School of Medicine, Nihon University, Tokyo 173-8610, Japan; 5 Department of Obstetrics and Gynecology, Graduate School of Medicine, The University of Tokyo, Tokyo 113-8654, Japan

**Keywords:** comprehensive genomic profiling, genomic landscape, tumor mutational burden, microsatellite instability, endometrial cancer, cervical cancer, ovarian cancer

## Abstract

**Simple Summary:**

This study comprehensively investigated the genomic landscape of >3000 gynecological malignancies (endometrial, cervical, and ovarian cancers) in Japan. The Center for Cancer Genomics and Advanced Therapeutics database used in this study is a useful tool containing real-world data of patients with poor prognoses, as comprehensive genomic profiling tests are limited to patients with cancer who have completed standardized treatments in Japan. Genomic profiling based on histological subtypes, tumor mutational burden, and microsatellite instability highlights actionable mutations for future drug development for each gynecological cancer.

**Abstract:**

This study aimed to comprehensively clarify the genomic landscape and its association with tumor mutational burden-high (TMB-H, ≥10 mut/Mb) and microsatellite instability-high (MSI-H) in endometrial, cervical, and ovarian cancers. We obtained genomic datasets of a comprehensive genomic profiling test, FoundationOne^®^ CDx, with clinical information using the “Center for Cancer Genomics and Advanced Therapeutics” (C-CAT) database in Japan. Patients can undergo the tests only after standardized treatments under universal health insurance coverage. Endometrial cancers were characterized by a high frequency of TMB-H and MSI-H, especially in endometrioid carcinomas. The lower ratio of *POLE* exonuclease mutations and the higher ratio of *TP53* mutations compared to previous reports suggested the prognostic effects of the molecular subtypes. Among the 839 cervical cancer samples, frequent mutations of *KRAS*, *TP53*, *PIK3CA*, *STK11*, *CDKN2A*, and *ERBB2* were observed in adenocarcinomas, whereas the ratio of TMB-H was significantly higher in squamous cell carcinomas. Among the 1606 ovarian cancer samples, genomic profiling of serous, clear cell, endometrioid, and mucinous carcinomas was characterized. Pathogenic mutations in the *POLE* exonuclease domain were associated with high TMB, and the mutation ratio was low in both cervical and ovarian cancers. The C-CAT database is useful for determining the mutational landscape of each cancer type and histological subtype. As the dataset is exclusively collected from patients after the standardized treatments, the information on “druggable” alterations highlights the unmet needs for drug development in major gynecological cancers.

## 1. Introduction

Comprehensive genomic profiling (CGP) tests broadly explore treatments based on individual genomic information [1]. Until June 2023, three CGP tests have been clinically applicable in Japan, including a tumor-only panel, the FoundationOne^®^ CDx (F1CDx) assay; a liquid biopsy panel, the FoundationOne Liquid^®^ CDx assay; and a tumor/normal paired panel, the OncoGuide^TM^ NCC Oncopanel System [2,3]. All genomic profiling data and clinical information are transferred to the Center for Cancer Genomics and Advanced Therapeutics (C-CAT) with written informed consent (agreement ratio, 99.7%), and the data are available for research use [3]. As the CGP tests under the universal health insurance system in Japan are only applicable to patients who have (already or almost) finished standardized treatments, the dataset is composed of patients with a poor prognosis for all cancer types. Liquid biopsy is limited to patients whose tissue specimens are not available or not suitable for CGP, and to date, F1CDx has been broadly tested (>75%) in Japan. The C-CAT database enables us to understand the mutational landscape, tumor mutational burden (TMB), and microsatellite instability (MSI) status of any type of advanced solid tumor [3].

Endometrial, cervical, and ovarian cancers are the major types of gynecological malignancies. Platinum-based chemotherapy is typically used for these three cancers, and CGP tests are anticipated to identify novel treatment options. In endometrial cancer, genomic alterations are common in the phosphatidylinositol-3 kinase (PI3K) pathway (such as *PTEN*, *PIK3CA*, and *PIK3R1*) and the receptor tyrosine kinase/RAS pathway [4,5]. Notably, four major molecular subtypes have been identified: (i) *POLE* ultramutated (in the exonuclease domain), (ii) MSI-high (hypermutated), (iii) copy number low (mainly endometrioid), and (iv) copy number-high (serous-like) [4,6]. Immunohistochemistry for mismatch repair (MMR) genes and TP53 can alternatively be considered MSI-high (MSI-H) and copy number-high, respectively [7]. In cervical cancer, genomic alterations in *PIK3CA* are the most common (26%), followed by *EP300* (11%) and *FBXW7* (11%) [8]. Genomic alterations in *BRCA1*/*2* (both germline and somatic) and *TP53* are common in high-grade serous ovarian carcinomas [9,10]. Genomic alterations of *ARID1A* and *PIK3CA* have been detected in 30–60% of endometriosis-associated ovarian carcinomas, that is, endometrioid and clear cell ovarian carcinomas [11]. Genomic alterations in *KRAS* and *BRAF* in the MAPK pathway and *TP53* are common in mucinous ovarian carcinomas [12].

Both MSI-high and TMB-high (TMB-H, ≥10 mutations/megabase [mut/Mb]) are used as companion diagnostics for an immune checkpoint inhibitor (ICI), pembrolizumab, in solid tumors [13,14]. In addition to these tumor-agnostic indications, since December 2022, cemiplimab monotherapy (anti-programmed cell death 1 antibody) has been approved in recurrent cervical cancer as a second-line or later treatment in Japan, regardless of PD-L1 status [15]. Since December 2021, lenvatinib (a multi-tyrosine kinase inhibitor) plus pembrolizumab has been approved in Japan for the treatment of advanced/recurrent endometrial cancer, regardless of MSI status [16]. Recently, ICI plus platinum-based chemotherapy has shown significantly better overall survival and/or progression-free survival in both endometrial and cervical cancers (either primary advanced or recurrent) [17,18,19]. However, the prognostic benefits of ICI-containing regimens are significantly greater in the presence of MSI-H and/or deficient MMR (dMMR) in endometrial cancer and PD-L1 markers in cervical cancer [17,18,19]. In ovarian cancer, TMB-H or MSI-H remains the only indication for pembrolizumab, although several ongoing clinical trials include ICIs [20].

In the present study, we aimed to focus on the mutational landscape, TMB, and MSI status of endometrial, cervical, and ovarian cancers in Japanese patients using the C-CAT database of F1CDx (registered from June 2019 to May 2022; https://www.ncc.go.jp/jp/c_cat/use/index.html, (accessed on 1 June 2022)).

## 2. Materials and Methods

### 2.1. Patient Samples of FoundationOne^®^ CDx (F1CDx) from the Center for Cancer Genomics and Advanced Therapeutics Database

**Figure 1:**
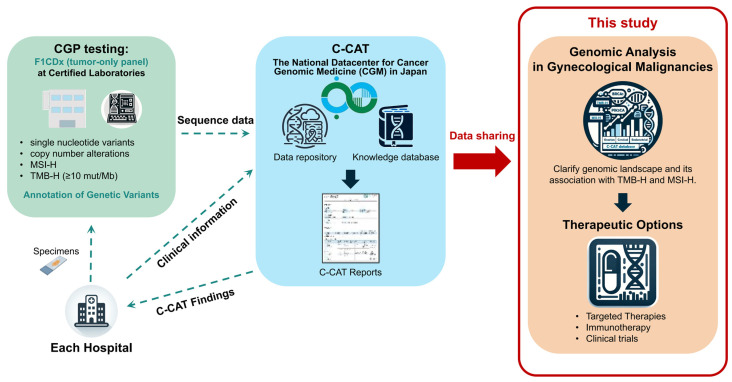
A workflow of the study to analyze the C-CAT database. Clinical information from each hospital and genomic data from certified laboratories (i.e., Foundation Medicine, Inc. for F1CDx) are collected and sent to the C-CAT data center. C-CAT reports with annotations of each alteration and therapeutic options are returned to each hospital. The C-CAT datasets with clinical information can be used for research purposes with the permission of institutional ethics committees and the Information Utilization Review Board of C-CAT.

This Japanese cohort study included 561 endometrial, 839 cervical, and 1606 ovarian cancers that were analyzed using F1CDx under health insurance coverage. The data were obtained from the C-CAT database organized by the National Cancer Center of Japan, which stores the CGP data tests [3]. The CGP tests in Japan are limited to patients with solid cancers who have finished (or are expected to finish) standard treatments for advanced unresectable diseases. Therefore, the patients enrolled generally had poor prognoses and were resistant to platinum-based chemotherapies for all three gynecological cancers. We logged into the C-CAT system to collect 3006 of 25,504 patients’ F1CDx data for the three gynecological cancers (between June 2019 and May 2022). We accessed the database on 1 June 2022. The workflow of this study is shown in Figure 1. The histological subtypes of each cancer are summarized in Supplementary Table S1. In this study, pure sarcomas were not included in endometrial cancer, whereas 2 sarcomas and 63 non-epithelial tumors were included in cervical and ovarian cancers, respectively. This study was approved by our institutional ethics committee (#2021341G) and the Information Utilization Review Board of C-CAT (#CDU2022-026N).

### 2.2. F1CDx Testing

F1CDx is a tumor-only panel using DNA isolated from formalin-fixed, paraffin-embedded tumor tissue specimens, which can detect substitutions, insertions, and deletions (indels); copy number alterations (CNAs) in 324 genes; gene rearrangements in 36 genes; and genomic signatures, including MSI and TMB [21]. MSI status is reported as “cannot be determined” when the quality is insufficient. TMB by F1CDx is determined by counting all synonymous and non-synonymous variants, except for hotspot genomic alterations, and is considered TMB-H when reported as ≥10 mut/Mb. In our study, all genetic variants, including single nucleotide variants, CNAs, and gene fusions, were annotated as pathogenic or likely pathogenic based on CIViC, BRCAExchange, ClinVar, and COSMIC [3]. MSI-H and TMB-H are tumor-agnostically approved as CDx for pembrolizumab in solid cancers in Japan. In this study, cases with “cannot be determined” for either TMB or MSI were excluded from the analysis (31 endometrial, 70 cervical, and 80 ovarian cancers).

### 2.3. Statistical Analyses and Graphical Representations

Quantitative variables were analyzed using one-way analysis of variance (ANOVA) (when normality was assumed) and the Kruskal–Wallis H test (when normality could not be assumed) for comparisons among the three groups. Pearson’s correlation test was used for correlation analysis between the two groups. All reported *p* values were two-tailed, and *p* < 0.05 was considered significant unless otherwise specified. All the graphs, calculations, and statistical analyses were performed using GraphPad Prism software 9.3.0 and R 4.2.0 software. The collation and visual analysis of alteration data were implemented using the “ComplexHeatmap” package in R.

## 3. Results

### 3.1. Genomic Alteration Profiles across Cancer Types

**Figure 2:**
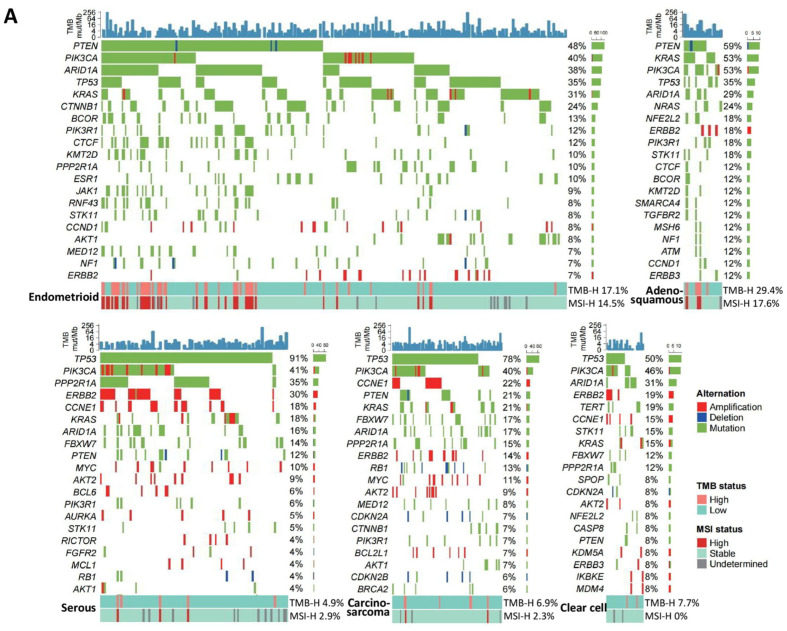

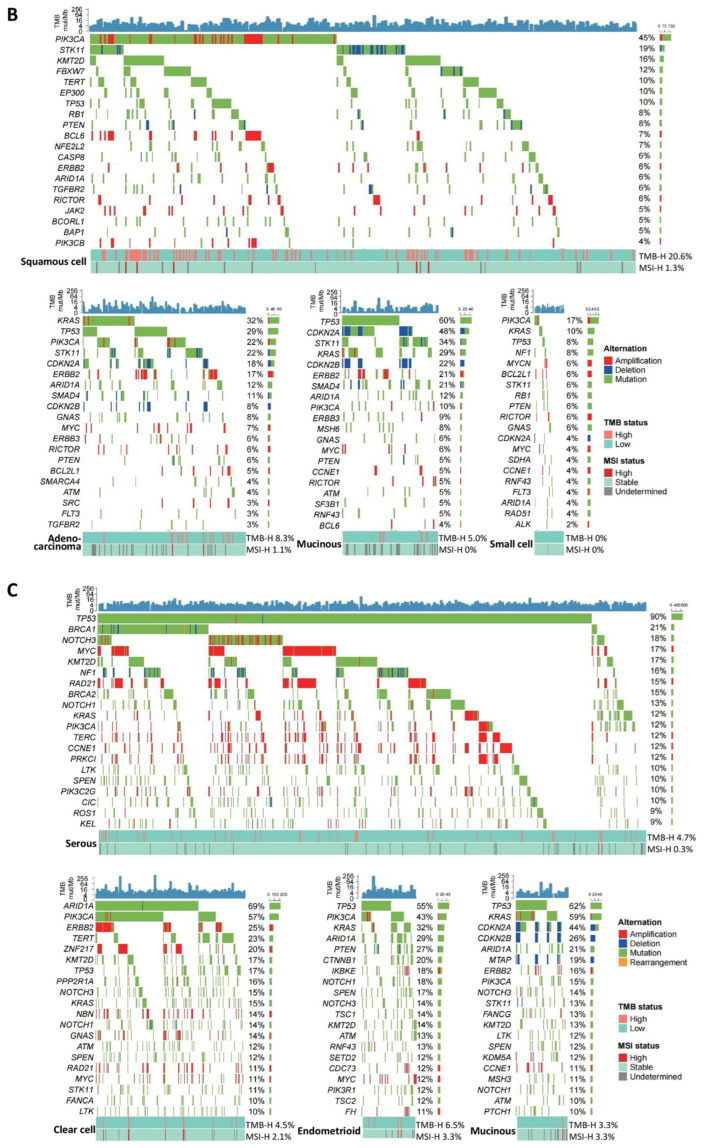
Genomic landscape of three gynecological cancers according to histological subtypes. Recurrently mutated genes (up to 20) with types of alterations are listed with the status of tumor mutational burden (TMB) and microsatellite instability in (**A**) endometrial, (**B**) cervical, and (**C**) ovarian cancers. The upper plot represents the TMB scores using FoundationOne^®^ CDx (registered from June 2019 to May 2022; https://www.ncc.go.jp/jp/c_cat/use/index.html, (accessed on 1 June 2022)).

We analyzed the genomic alterations (pathogenic or likely pathogenic) in F1CDx from the C-CAT database in 561 endometrial, 839 cervical, and 1606 ovarian cancer samples. The mutational landscape of frequently mutated (pathogenic or likely pathogenic) genes (top 30) in each cancer type and histological subtype is summarized in Supplementary Figure S1, and Figure 2, respectively (A: endometrial, B: cervical, and C: ovarian cancers).

#### 3.1.1. Endometrial Cancer

Genomic alterations were common in *TP53* (*n* = 305, 54.4%), *PIK3CA* (*n* = 231, 41.2%), *PTEN* (*n* = 194, 34.6%), *ARID1A* (*n* = 172, 30.7%), and *KRAS* (*n* = 146, 26.0%) (Supplementary Figure S1A). The ratio of *TP53* (*p* < 0.001) was significantly higher, and the ratios of *PTEN* (*p* < 0.001) and *PIK3CA* (*p* = 0.0028) were significantly lower in the C-CAT database compared with The Cancer Genome Atlas (TCGA) database. In addition, the ratio of pathogenic/likely pathogenic alterations in *POLE* in the exonuclease domain was only 1.4% (7.3% in the TCGA), supporting the favorable prognosis of *POLE-*mutated endometrial carcinomas [4].

**Figure 3:**
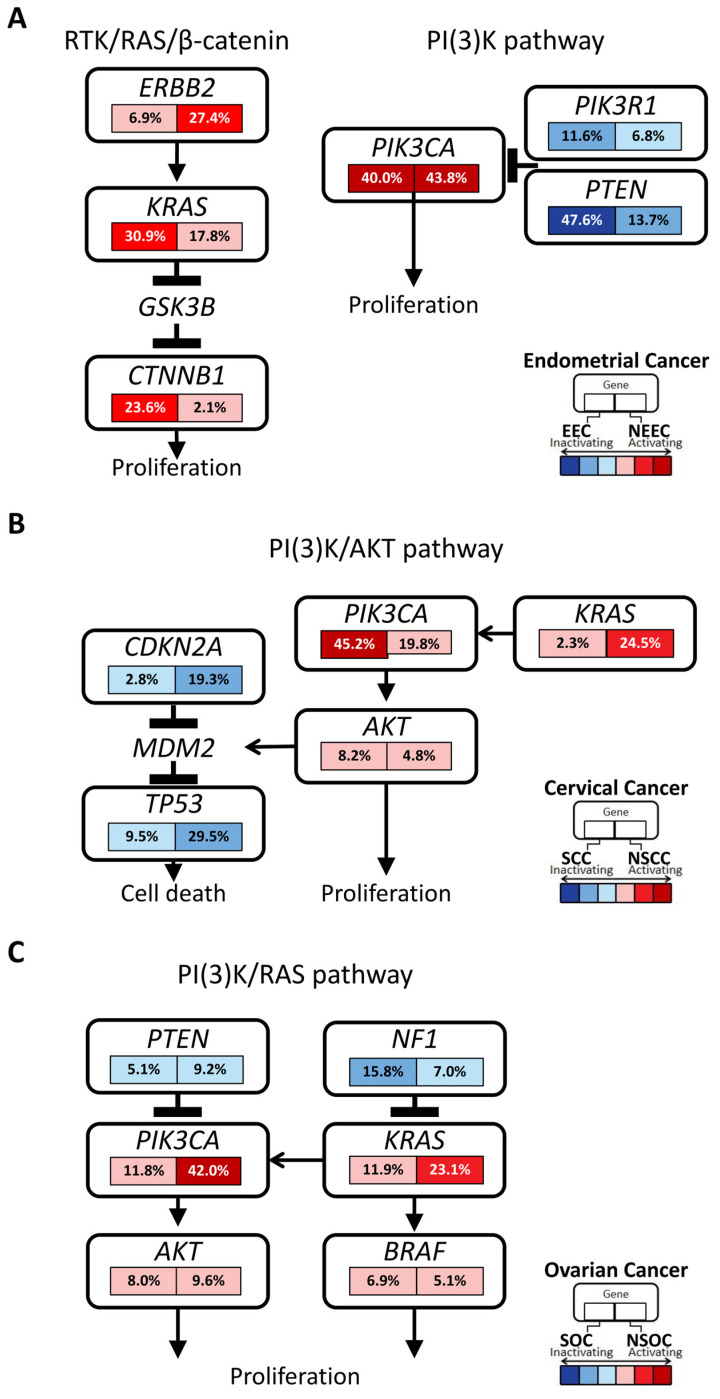
Frequency of genomic alterations in the key signaling pathways (mainly focusing on the KRAS and PI3K pathways), according to major histological types in (**A**) endometrial, (**B**) cervical, and (**C**) ovarian cancers. EEC, endometrioid endometrial carcinoma; NEEC, non-endometrioid endometrial carcinoma; SCC, squamous cell carcinoma; NSCC, non-squamous cell carcinoma; SOC, serous ovarian carcinoma; NSOC, non-serous ovarian carcinoma.

Endometrioid endometrial carcinoma, accounting for 49.0% of our study, was characterized by genomic alterations of *PTEN* (47.6% vs. 13.7%, *p* < 0.001), *KRAS* (30.9% vs. 17.8%, *p* = 0.0037), *CTNNB1* (23.6% vs. 2.1%, *p* < 0.001), and *ARID1A* (37.8% vs. 22.6%, *p* = 0.0015), compared with non-endometrioid endometrial carcinomas (serous, clear cell, and mixed carcinomas) (Figure 2A). The high frequency of *PIK3CA* genomic alterations, regardless of the histological types, suggested the need for potential therapies targeting the PI3K pathway (Figure 3A and Table 1).

Genomic alterations of both *TP53* (80.8% vs. 35.3%, *p* < 0.001) and *ERBB2* (27.4% vs. 6.9%, *p* < 0.001) were more frequent in non-endometrioid carcinomas (Figure 3A).

#### 3.1.2. Cervical Cancer

Among the 839 samples, genomic alterations of *PIK3CA* were the most prevalent (*n* = 270, 32.2%), followed by *STK11* (*n* = 170, 20.3%), *TP53* (*n* = 166, 19.8%), *KRAS* (*n* = 117, 13.9%), and *CDKN2A* (*n* = 96, 11.4%) (Supplementary Figure S1B). *ERBB2* genomic alterations were observed at 9.7% (amplifications at 6.3% and pathogenic variants at 4.1%), which might lead to clinical trials (Table 1). Squamous cell carcinomas (*n* = 389) exhibited a significantly higher *PIK3CA* mutation rate of 45.2% compared with 19.8% in non-squamous cell carcinomas (*n* = 420) (Figure 3B). In adenocarcinomas (*n* = 180), *KRAS* genomic alterations were most frequently observed (32.2%), followed by *TP53* (29.4%), *PIK3CA* (22.2%), *STK11* (22.2%), *CDKN2A* (18.3%), *ERBB2* (16.7%), and *ARID1A* (11.7%) (Figure 2B).

#### 3.1.3. Ovarian Cancer

Among the 1606 samples, *TP53* genomic alterations (*n* = 1054, 65.6%) were the most frequent, followed by *ARID1A* (*n* = 407, 25.3%), *PIK3CA* (*n* = 406, 25.3%), *KRAS* (*n* = 272, 16.9%), *KMT2D* (*n* = 272, 16.9%), and *NOTCH3* (*n* = 270, 16.8%) (Supplementary Figure S1C and Table 1).

In serous carcinomas, genomic alterations of *BRCA1* and *BRCA2* accounted for 21.2% (166/784) and 14.7% (115/784) of cases, respectively (Figure 2C). The coexistence rate of these two alterations was 4.8% (38/784), which was significantly higher than those reported by 0.6% (2/316) [12] and 0% (0/205) [30]. Genomic alterations in other homologous recombination repair genes included *ATM* (8.8%), *PALB2* (7.1%), and *CDK12* (6.6%) (Figure 2C). Genomic alterations in *TP53*, *NF1, KRAS*, and *PIK3CA* were detected in 90.4% (*n* = 709), 15.8% (*n* = 124), 11.9% (*n* = 93), and 11.7% (*n* = 92) of cases, respectively (Figure 3C).

Clear cell carcinomas were examined in 20.7% (*n* = 333) of the cases, with genomic alterations in *ARID1A* (*n* = 231, 69.4%) and *PIK3CA* (*n* = 190, 57.1%), consistent with previous reports [15] (Figure 2C). Genomic alterations of *TP53* were observed in 16.5% (*n* = 55) of the cases and were negatively associated with alterations in both *ARID1A* (*p* < 0.001) and *PIK3CA* (*p* < 0.001) (Figure 2C). Genomic alterations of *ERBB2* (primarily amplification) and *KRAS* were detected in 25% and 15% of the cases, respectively.

In endometrioid carcinomas, the ratios of genomic alterations in *TP53*, *PIK3CA*, *KRAS ARID1A*, *PTEN,* and *CTNNB1* were 55.4%, 43.5%, 31.5%, 29.3%, 27.2%, and 19.6%, respectively. *TP53* alterations were negatively associated with alterations in *ARID1A* (*p* = 0.0006), *KRAS* (*p* = 0.0017), *PTEN* (*p* = 0.0002), and *CTNNB1*(*p* < 0.001).

In mucinous carcinomas, genomic alterations of *TP53*, *KRAS*, *CDKN2A*, and *CDKN2B* were detected in 61.5%, 59.3%, 44.0%, and 26.4% of the cases, respectively. Although genomic alterations of *BRAF* were approximately 20% [16], the ratio was only 5.5% (*n* = 5) in this study. Genomic alterations in *ERBB2* were detected in 16.5% of the cases.

### 3.2. Prevalence of Microsatellite Instability-High (MSI-H) and Tumor Mutation Burden-High (TMB-H)

**Figure 4:**
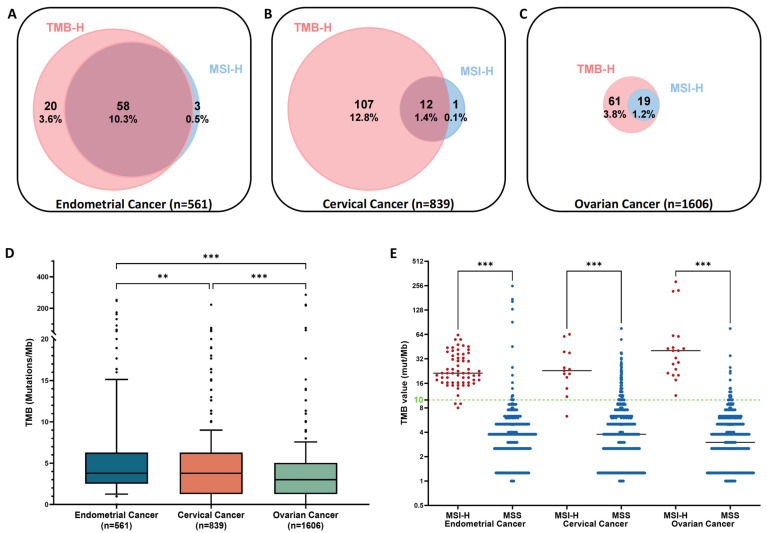
Venn diagrams of tumor mutational burden (TMB)-high and microsatellite instability-high in (**A**) endometrial, (**B**) cervical, and (**C**) ovarian cancers. (**D**) Box plots of TMB levels and (**E**) scatter dot plots of TMB distribution according to the MSI status in each cancer. ** *p* < 0.01, *** *p* < 0.001 using one-way analysis of variance with the Kruskal–Wallis test.

Among the 561 endometrial cancer samples, 78 (13.9%) were TMB-H and 61 (10.9%) were MSI-H. A total of 58 of the 61 MSI-H tumors were TMB-H, whereas 20 of the 78 (25.6%) TMB-H tumors were non-MSI-H tumors (Figure 4A).

Among the 839 cervical cancer samples, 119 (14.2%) and 13 (1.5%) were TMB-H and MSI-H, respectively (Figure 4B). Only 1 of 13 (7.7%) cervical cancers with MSI-H was TMB-low (TMB-L) (Figure 4B). Among the 1606 ovarian cancer samples, 80 (5.0%) were MSI-H and 19 (1.2%) were TMB-H (Figure 4C). All 19 MSI-H ovarian cancer samples were classified as TMB-H (Figure 4C).

The TMB value in endometrial cancer was significantly higher than that in cervical cancer (*p* < 0.001 by one-way ANOVA with the Kruskal–Wallis test) and ovarian cancer (*p* < 0.001) (Figure 4D). The median TMB values in MSI-H tumors were 21.4 mut/Mb in endometrial, 23.0 mut/Mb in cervical, and 40.4 mut/Mb in ovarian cancers (Figure 4E), with a strong correlation between MSI and TMB in these three cancer types (*p* < 0.001) (Figure 4E).

The TMB and MSI statuses were distinct among the histological subtypes of each cancer (Supplementary Table S2).

**Figure 5:**
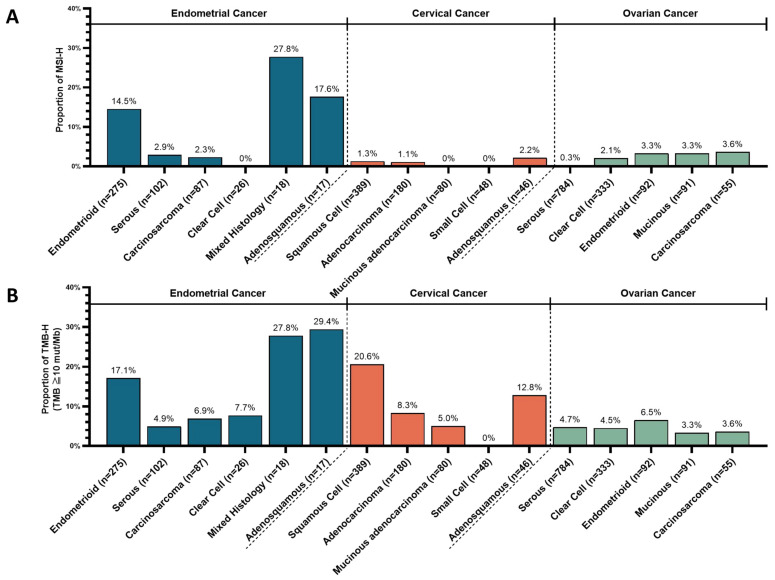
Frequency of (**A**) microsatellite instability-high and (**B**) tumor mutational burden-high according to the major histological subtypes in endometrial, ovarian, and cervical cancers.

In endometrial cancer, the MSI-H ratio was significantly higher in endometrioid carcinomas (40/275, 14.5%) compared to serous carcinomas, clear cell carcinomas, and carcinosarcomas (5/215, 2.3%) (*p* < 0.001) (Figure 5A).

In cervical cancer, the MSI-H ratio was not significantly different between squamous cell carcinomas (1.3%) and adenocarcinomas (1.1%) (Figure 5A). In ovarian cancer, the MSI-H ratio was <4.0% in all histological subtypes and was significantly lower in serous carcinomas (2/784, 0.3%) compared with non-serous carcinomas (15/571, 2.6%) (*p* = 0.0002) (Figure 5A). In endometrial cancer, the ratio of TMB-H was high in adenosquamous carcinomas (5/17, 29.4%), mixed carcinomas (5/18, 27.8%), and endometrioid carcinomas (47/275, 17.1%), whereas it was only 4.9–7.7% in serous carcinomas, clear cell carcinomas, and carcinosarcomas (Figure 5B). In cervical cancer, the TMB-H ratio was significantly higher in squamous cell carcinomas (80/389, 20.6%) compared with adenocarcinomas (8.3%, 15/180) and mucinous carcinomas (5.0%, 4/80) (*p* = 0.0002 and *p* = 0.0004, respectively) (Figure 5B). In ovarian cancer, the TMB-H ratio was 3.3–6.5% in all histological subtypes.

### 3.3. Mismatch Repair-Related Mutations in Tumors with MSI-H and TMB-H

We analyzed the correlation between genomic alterations in MMR genes (dMMR, defined as genomic alterations in *MLH1*, *PMS2*, *MSH2*, and *MSH6*) and the MSI status. In endometrial cancer, the dMMR ratio was 31.1% (19/61) in MSI-H, which was significantly higher than the 2.8% (13/469) reported in microsatellite stable (MSS) tumors (*p* < 0.001) (Supplementary Figure S2A). The dMMR ratios in MSI-H and MSS in cervical cancer were 61.5% (8/13) and 4.6% (35/756) (*p* < 0.001), respectively, whereas those in ovarian cancer were 84.2% (16/19) and 13.5% (203/1507) (*p* < 0.001), respectively (Supplementary Figure S2A).

Next, we analyzed the dMMR ratio in MSS tumors. The dMMR ratio was significantly higher in TMB-H tumors (25%) compared with TMB-L tumors (2.5%) in MSS endometrial cancer (*p* = 0.0003) (Supplementary Figure S2B). In MSS cervical cancer, dMMR was also more frequent in TMB-H (9.4%) compared with TMB-L (4.3%) (*p* = 0.0302). No statistically significant difference was detected in ovarian cancer (22.4% vs. 13.5%, *p* = 0.0769) (Supplementary Figure S2B).

The highest prevalence of genomic alterations in MSI-H endometrial cancer was observed in *MSH6* (*n* = 14, 23.0%), followed by *MSH2* (*n* = 8, 13.1%), *MLH1* (*n* = 4, 6.6%), and *PMS2* (*n* = 1, 1.6%) (Supplementary Table S3). Similarly, this prevalence was confirmed in ovarian cancer with MSI-H, with genomic alteration rates of *MSH6*, *MSH2*, *MLH1*, and *PMS2* of 52.6%, 36.8%, 31.6%, and 10.5%, respectively. In MSI-H cervical cancer, the genomic alteration rates of *MSH6* and *MLH1* were the highest (*n* = 4, 30.8%) (Supplementary Table S3).

### 3.4. Distribution of POLE Genomic Alterations in the Exonuclease Domain among TMB-H and Microsatellite Stable Subsets

All *POLE* variants (including variants of unknown significance [VUS]) are listed in Table 2.

The ultramutated genotype (TMB > 100 mut/Mb) was identified in eight tumors (five endometrial and three ovarian cancers). In endometrial cancer, all eight (1.4%) *POLE* exonuclease-mutated tumors were TMB-H (median TMB, 90.78 mut/Mb), of which only one was MSI-H (Table 2). Three MSI-H and TMB-H tumors showed VUS of *POLE* outside the exonuclease domain, which should be categorized as MSI-H, not as a *POLE* subgroup (Table 2). Pathogenic/likely pathogenic variants in the *POLE* exonuclease domain were detected in one case (0.12%) of cervical cancer and three cases (0.19%) of ovarian cancer. None of the *POLE* variants outside the exonuclease domain were annotated as pathogenic or likely pathogenic (Table 2).

### 3.5. Correlation among Genomic Alterations, MSI, and TMB

Finally, we focused on the mutational landscape of “TMB-H with MSS” and “MSI-H” tumors in each cancer type. The most frequent genomic alteration in the “MSI-H” group was *ARID1A* in all three cancer types. The ratios were 96.7% (59/61) in endometrial, 76.9% (10/13) in cervical, and 89.5% (17/19) in ovarian cancers (Supplementary Figure S3A–C). *PTEN* was another MSI-H-related gene. The ratios of *PTEN* alterations in the “MSI-H” group were 85.2% (52/61) in endometrial, 69.2% (9/13) in cervical, and 57.9% (11/19) in ovarian cancers, whereas the ratios of *PTEN* alterations in the “MSS with TMB-L” group were 28.2% (127/451) in endometrial, 7.5% (49/650) in cervical, and 6.3% (92/1449) in ovarian cancers.

In “TMB-H with MSS” tumors, the ratio of genomic alterations in *PIK3CA* was the most or the second highest, which was 61.1% in endometrial, 51.4% in cervical, and 31.0% in ovarian cancers (Supplementary Figure S3). Genomic alterations of *TP53* were most common in the TMB-H with MSS group in endometrial (61.1%) and ovarian (82.8%) cancers, whereas the rate was 12.0% in cervical cancer (usually human papillomavirus [HPV], which relates to the impairment of TP53 by the ubiquitin–proteasome pathway). The ratio of genomic alterations in *CDKN2A* and *CDKN2B* was also high in endometrial and ovarian cancers (Supplementary Figure S3).

## 4. Discussion

In this study, we analyzed 3006 endometrial, cervical, and ovarian cancers using a tumor-only panel, F1CDx. The Japanese CGP test dataset is unique in terms of eligible patients and insurance coverage. All the patients have finished or are expected to finish the standardized treatments and take the CGP tests under universal health insurance coverage [3,31]. Thus, any poor prognosis in Japanese patients with cancer may allow them to undergo CGP tests. Furthermore, a sufficient number of tumor specimens are usually available through surgery and/or biopsy. Therefore, the C-CAT database is suitable for analyzing the genomic profiles of patients with gynecological cancer with a poor prognosis.

In endometrial cancer, a comparison with the TCGA database highlighted the high incidence of genomic alterations of *TP53* (54.4%) and the low incidence of genomic alterations of *POLE* (1.4%) in this database. This discrepancy supports the significance of the molecular classification of “Proactive Molecular Risk Classifier for Endometrial Cancer” in endometrial cancer by POLE, dMMR, and TP53 [32]. Drug development is highly warranted in genomic alterations of the PI3K (*PTEN* and *PIK3CA*), RAS (*KRAS*), and wnt/β-catenin (*CTNNB1*) pathways in endometrioid carcinomas and *TP53*, *ERBB2*, and *PIK3CA* in non-endometrioid carcinomas (Table 1). A WEE1 inhibitor, adavosertib, showed an objective response rate of 29.4% in recurrent uterine serous carcinomas (usually *TP53* mutated), and an international phase IIb study is ongoing [33,34]. Further development of precision medicine in endometrial cancer is warranted.

In cervical cancer, the C-CAT dataset was helpful for elucidating the genomic profiling of adenocarcinomas, as the ratio of non-squamous cell carcinomas was significantly lower in the TCGA dataset (19.1%) than in the C-CAT database (53.6%) [8]. Key molecular targets, especially in adenocarcinomas, include *KRAS*, *ERBB2*, and *ARID1A.* According to the recently published 5th edition of the World Health Organization classification, cervical cancer is classified as HPV-associated and HPV-independent for each histological type [35]. As both the TP53 and RB pathways are impaired by HPV-E6 and HPV-E7 oncoproteins, respectively, genomic alterations of *TP53*, *RB*, and *CDKN2A*/*2B* are informative for speculating HPV-independent cervical cancers, especially in gastric-type mucinous adenocarcinomas [36,37].

One limitation of the C-CAT database is that data on low-grade serous ovarian carcinomas are mixed with those on high-grade serous carcinomas. Genomic alterations of *TP53* in 90% of serous carcinomas suggest that these tumors represent high-grade serous carcinomas. The RAS-MAPK signaling pathway (genomic alterations of *NF1* at 16% and *KRAS* at 12% with mutual exclusivity), the PI3K-mTOR pathway (*PIK3CA* at 12% and *TSC2* at 8%), and certain receptor tyrosine kinases (*ROS1* at 9% and *ERBB2* at 8%) might be candidates for targeted therapy in serous carcinomas. The pathogenicity of each alteration, especially in *BRCA1* and *BRCA2*, should be carefully addressed [9,30]. Drug development targeting *ARID1A* and *PIK3CA* in clear-cell ovarian carcinomas is also warranted. Currently, a p110alpha selective inhibitor, CYH33, is under phase 2 clinical trials (NCT05043922, jRCT2031210216), which recruits patients with clear cell ovarian carcinoma with hotspot mutations in *PIK3CA* (Table 1) [29]. Targeting the RAS-MAPK pathway should be key in mucinous carcinomas.

Candidate tumor-agnostic molecular targets in the three gynecological malignancies included *ERBB2*, *PIK3CA*, *ARID1A*, and *KRAS*. An antibody-drug conjugate, trastuzumab deruxtecan, showed an overall response rate of 54.5–70.0% in endometrial carcinosarcomas positive for HER2 in the STATICE trial [28]. Genomic alterations in *ARID1A* may lead to novel molecular-targeted therapies, including an EZH2 inhibitor and an enzyme for antioxidant glutathione synthesis (Table 1) [23,24]. p110alpha selective inhibitors (alpelisib), KRAS^G12C^ inhibitors (sotorasib), KRAS^G12D^ degraders (ASP3082), and a CBP/β-catenin inhibitor (E7386) may be candidates [22,25,26,27]. The Japanese Gynecologic Oncology Group is currently conducting a basket trial on niraparib monotherapy for any gynecological cancer (except ovarian cancer) with *BRCA1*/*2* genomic alterations, which targets a rare fraction of each cancer type [38].

In agreement with previous findings, MSI-H in this study was the main causative genomic finding for TMB-H induction in endometrial cancer, whereas it shared only 10% and 24% of TMB-H in cervical and ovarian cancers, respectively [39,40]. A low TMB-H ratio (5.0%) in ovarian cancer may be associated with limited sensitivity to ICIs [41]. A comparison between “TMB-H with MSS” and “MSI-H” in each cancer type is informative to elucidate real “driver” alterations. In endometrial and ovarian cancers, the frequency of genomic alterations in *TP53* and *CDKN2A*/*2B* was significantly higher in the group of “TMB-H with MSS”. These findings suggest that TMB-H should be subclassified according to the MSI status. Although pembrolizumab has been approved in any solid cancers with either TMB-H and MSI-H, combination therapies with immune checkpoint inhibitors may be developed separately according to the status of TMB and MSI.

This study has some limitations. First, CGP tests in Japan are reimbursed only for patients who have (almost) finished standardized treatments, suggesting that patients with rapid progression may miss the opportunity to undergo CGP tests. In addition, this study lacks data from patients without medical insurance due to the universal health insurance system in Japan. Second, the response to genome-matched therapies was not analyzed in this study because of the low accessibility of the recommended drugs. Third, the C-CAT database was deposited at designated hospitals located in Japan. Therefore, most of the patients were Japanese.

## 5. Conclusions

This study uniquely illustrates the genomic landscape of three major gynecological cancers in the Japanese cohort. It highlights the necessity of future drug developments in each cancer type and each histological subtype. *ERBB2*, *PIK3CA*, *ARID1A*, and *KRAS* would be key molecular targets in gynecological cancers. Furthermore, the prevalence and correlation between TMB and MSI may influence future immunotherapy, including combination therapies. These insights reinforce the necessity of molecular classification in understanding tumor biology and developing personalized therapies, underlining the potential of genomic profiling in precision oncology.

## Figures and Tables

**Table 1:** Genomic Alterations and Potential Targeted Therapies in Gynecological Cancers.

Cancer Type	Genomic Alteration	Frequency	Histological Type	Potential Targeted Therapies	References
Endometrial	*TP53*	54.4%	Serous (91%); Carcinosarcoma (78%); Clear cell (50%), etc.	-	-
	*PIK3CA*	41.2%	Adenosquamous (53%); Clear cell (46%); Serous (41%), etc.	p110alpha selective inhibitor (alpelisib)	[22]
	*PTEN*	34.6%	Adenosquamous (59%); Endometrioid (48%); Carcinosarcoma (21%), etc.	-	-
	*ARID1A*	30.7%	Endometrioid (38%); Clear cell (31%); Adenosquamous (29%), etc.	EZH2 inhibitor; Enzyme for antioxidant glutathione synthesis	[23,24]
	*KRAS*	26.0%	Adenosquamous (53%); Endometrioid (31%); Carcinosarcoma (21%), etc.	KRAS-G12C inhibitors (sotorasib); KRAS-G12D degrader (ASP3082)	[25,26]
	*CTNNB1*	15.0%	Endometrioid (24%), etc.	CBP/β-catenin inhibitor (E7386)	[27]
	*ERBB2*	14.0%	Serous (30%); Clear cell (19%); Adenosquamous (18%), etc.	Trastuzumab deruxtecan	[28]
	TMB-H	13.9%	Adenosquamous (29%); Endometrioid (17%); Clear cell (8%), etc.	Pembrolizumab; Lenvatinib plus pembrolizumab (regardless of MSI status); Pembrolizumab plus chemotherapy; Dostarlimab	[13,14,16,17,18]
	MSI-H	10.9%	Adenosquamous (18%); Endometrioid (15%), etc.		
Cervical	*PIK3CA*	32.2%	Squamous cell (45%); Adenocarcinoma (22%); Small cell (17%), etc.	p110alpha selective inhibitor (alpelisib)	[22]
	*STK11*	20.3%	Mucinous (34%); Adenocarcinoma (22%); Squamous cell (19%), etc.	-	-
	*TP53*	19.8%	Mucinous (60%); Adenocarcinoma (29%); Squamous cell (10%), etc.	-	-
	*KRAS*	13.9%	Adenocarcinoma (32%); Mucinous (29%); Small cell (10%), etc.	KRAS-G12C inhibitors (sotorasib); KRAS-G12D degrader (ASP3082)	[25,26]
	*CDKN2A*	11.4%	Mucinous (48%); Adenocarcinoma (18%) Small cell (4%), etc.	-	-
	TMB-H	14.2%	Squamous cell (21%); Adenocarcinoma (8%), etc.	Pembrolizumab	[13,14]
	MSI-H	1.5%	Adenocarcinoma (2%), etc.		
Ovarian	*TP53*	65.6%	Serous (90%); Mucinous (62%); Endometrioid (55%), etc.	-	-
	*BRCA1*/ *BRCA2*	25.4%	Serous (33%); Endometrioid (18%); Mucinous and Clear cell (13%), etc.	PARP inhibitors	-
	*ARID1A*	25.3%	Clear cell (69%); Endometrioid (29%); Mucinous (21%), etc.	EZH2 inhibitor; Enzyme for antioxidant glutathione synthesis	[23,24]
	*PIK3CA*	25.3%	Clear cell (57%); Endometrioid (43%); Mucinous (15%), etc.	p110alpha selective inhibitors (CYH33; alpelisib)	[22,29]
	*KRAS*	16.9%	Mucinous (59%); Endometrioid (32%); Clear cell (15%), etc.	KRAS-G12C inhibitors (sotorasib); KRAS-G12D degrader (ASP3082)	[25,26]
	TMB-H	5.0%	-	Pembrolizumab	[13,14]
	MSI-H	1.2%	-		

**Table 2:** List of *POLE* variants with their pathogenicity, MSI, and TMB status in each cancer.

**Endometrial Cancer (*n* = 561)**							
	**Histology**	**Gene**	**Variants**	**Clinical Significance**	**MSI Status**	**TMB Status**	**TMB Value (mut/Mb)**
**Exonuclease Domain (*n* = 8; 1.4%)**					**MSI-H 12.5%**	**TMB-H** **100%**	**Median** **146.89**
1	Endometrioid	POLE	P286R	Likely pathogenic	Stable	high	253.4
2	Endometrioid	POLE	P286R	Likely pathogenic	Undetermined	high	247.1
3	Adenosquamous	POLE	V411L	Likely pathogenic	Stable	high	174.0
4	Carcinosarcoma	POLE	P286R	Likely pathogenic	Stable	high	162.7
5	Endometrioid	POLE	P286R	Likely pathogenic	Stable	high	131.1
6	Endometrioid	POLE	P286R	Likely pathogenic	Stable	high	90.8
7	Endometrioid	POLE	V411L	Likely pathogenic	Stable	high	25.2
8	Adenosquamous	POLE	W243 *	Pathogenic	High	high	18.9
**Non-Exonuclease Domain (*n* = 3; 0.5%)**					**MSI-H** **100%**	**TMB-H** **100%**	**Median** **30.26**
1	Endometrioid	POLE	K1170fs*49		High	high	20.2
2	Endometrioid	POLE	P172fs*3		High	high	36.6
3	Endometrioid	POLE	S2173fs*130		High	high	30.3
**Cervical Cancer (*n* = 839)**							
	**Histology**	**Gene**	**Variants**	**Clinical Significance**	**MSI Status**	**TMB Status**	**TMB Value (mut/Mb)**
**Exonuclease Domain (*n* = 1; 0.1%)**					**MSI-H** **100%**	**TMB-H** **100%**	**Median** **23.0**
1	Squamous Cell	POLE	L283fs*5	Pathogenic	high	high	23.0
**Non-Exonuclease Domain (*n* = 6; 0.7%)**					**MSI-H** **0%**	**TMB-H** **66.7%**	**Median** **11.81**
1	Squamous Cell	POLE	E179 *		stable	high	22.7
2	Squamous Cell	POLE	E586 *		stable	high	17.7
3	Squamous Cell	POLE	Q670 *		stable	high	12.6
4	Squamous Cell	POLE	E1085K		stable	high	11.0
5	Adenocarcinoma	POLE	E18K		stable	low	7.6
6	Adenocarcinoma	POLE	R1077fs*43		stable	low	1.3
**Ovarian Cancer (*n* = 1606)**							
	**Histology**	**Gene**	**Variants**	**Clinical Significance**	**MSI Status**	**TMB Status**	**TMB Value (mut/Mb)**
**Exonuclease Domain (*n* = 4; 0.2%)**					**MSI-H** **50%**	**TMB-H** **75%**	**Median** **146.89**
1	Endometrioid	POLE	V411L	Likely pathogenic	high	high	223.2
2	Clear cell	POLE	T278K	Pathogenic	high	high	218.1
3	Unknown	POLE	P286R	Likely pathogenic	stable	high	75.7
4	Serous	POLE	L432V		stable	low	5.0
**Non-Exonuclease Domain (*n* = 64; 4.0%)**					**MSI-H** **9.4%**	**TMB-H** **17.2%**	**Median** **3.78**
1	Clear cell	POLE	H1356R; N1369S		high	high	286.0
2	Unknown	POLE	c.4551 + 2_4551 + 3 delTG		high	high	61.8
3	Clear cell	POLE	W1251 *		high	high	60.5
4	Carcinosarcoma	POLE	T41M		high	high	42.9
5	Mucinous	POLE	L1983fs*76		high	high	29.0
6	Carcinosarcoma	POLE	V1368M; V1929fs*70		high	high	17.7
7	Others	POLE	D934Y		stable	high	22.7
8	Endometrioid	POLE	R847L		stable	high	11.0
9	Clear cell	POLE	R2131C		stable	high	10.1
10	Serous	POLE	T880L		stable	high	10.1
11	Serous	POLE	N1521S		stable	high	10.1
12	Serous	POLE	G2046R		stable	low	9.0
13	Serous	POLE	R1059H		stable	low	8.0
14	Serous	POLE	F695L		stable	low	8.0
15	Serous	POLE	A1854S		stable	low	7.6
16	Serous	POLE	L53V		stable	low	7.6
17	Serous	POLE	amplification		stable	low	6.3
18	Clear cell	POLE	A2192V		stable	low	6.3
19	Unknown	POLE	A1778T		stable	low	6.3
20	Serous	POLE	I238F		stable	low	6.0
21	Serous	POLE	L1245V		stable	low	6.0
22	Serous	POLE	E2155Q		stable	low	6.0
23	Serous	POLE	I622M		stable	low	5.0
24	Unknown	POLE	T737A		stable	low	5.0
25	Serous	POLE	K1877E		stable	low	5.0
26	Clear cell	POLE	Q394 *		stable	low	5.0
27	Clear cell	POLE	R1382C		stable	low	4.0
28	Endometrioid	POLE	R1284Q		stable	low	3.8
29	Serous	POLE	L32P		stable	low	3.8
30	Serous	POLE	D934G		stable	low	3.8
31	Unknown	POLE	S1598C		stable	low	3.8
32	Endometrioid	POLE	Q394fs*18		stable	low	3.8
33	Serous	POLE	R1324H		stable	low	3.8
34	Endometrioid	POLE	V1736I		stable	low	3.8
35	Clear cell	POLE	T1196M		stable	low	3.0
36	Clear cell	POLE	V1512I		stable	low	2.5
37	Serous	POLE	amplification		stable	low	2.5
38	Clear cell	POLE	R847Q		stable	low	2.5
39	Unknown	POLE	E1554K		stable	low	2.5
40	Serous	POLE	A1140T		stable	low	2.5
41	Serous	POLE	D1516G		stable	low	2.5
42	Clear cell	POLE	S171F		stable	low	2.5
43	Granulosa cell	POLE	A788V		stable	low	2.5
44	Clear cell	POLE	G702R		stable	low	2.5
45	Serous	POLE	C2187Y		stable	low	2.5
46	Serous	POLE	R1077fs*43		undetermined	low	2.5
47	Endometrioid	POLE	F1435L		stable	low	1.3
48	Serous	POLE	T1666R		stable	low	1.3
49	Serous	POLE	T1196M		stable	low	1.3
50	Serous	POLE	R1284Q		stable	low	1.3
51	Serous	POLE	I218M		stable	low	1.3
52	Serous	POLE	R1485C		stable	low	1.3
53	Clear cell	POLE	T1313M		stable	low	1.3
54	Unknown	POLE	G541R		stable	low	1.3
55	Serous	POLE	T41M		stable	low	1.3
56	Unknown	POLE	R1284W		stable	low	1.3
57	Serous	POLE	L3V		stable	low	1.0
58	Mucinous	POLE	E1964D		stable	low	0.0
59	Clear cell	POLE	R1382C		stable	low	0.0
60	Mucinous	POLE	T1196M		stable	low	0.0
61	Clear cell	POLE	D1700V		stable	low	0.0
62	Serous	POLE	R1382C		stable	low	0.0
63	Clear cell	POLE	A1778T		stable	low	0.0
64	Clear cell	POLE	A1260T		undetermined	low	0.0

Blank: Pathogenicity is not defined in the C-CAT database. *: Genetic mutation notation indicates a stop codon, leading to premature termination of the protein. This results in a truncated protein with potential functional alterations or loss.

## Data Availability

https://www.ncc.go.jp/jp/c_cat/use/index.html (accessed on 1 June 2022).

## Supplementary Materials

The following supporting information can be downloaded at: https://www.mdpi.com/article/10.3390/cancers16010136/s1, Figure S1. Genomic landscape of three gynecological cancers. Recurrently mutated genes are listed with the status of TMB and MSI and with information about types of alterations and histological subtypes in (A) endometrial, (B) cervical, and (C) ovarian cancers. The upper plot represents the TMB scores by F1CDx.Waterfall plot of genetic alteration profiles in endometrial (A), cervical (B), and ovarian cancer (C). Figure S2. Frequency of genomic alterations in the mismatch repair (MMR) genes according to the MSI and TMB stauts in each cancer type. (A) Frequency of MMR alterations according to the MSI status, (B) Frequency of MMR alterations according to the TMB status. Comparisons between the groups were performed by Fisher’s exact test (* *p* < 0.05; ** *p* < 0.01; *** *p* < 0.001). MMR-pv, pathogenic variants in MMR genes, MMR-wt, no pathogenic variants (wild-type) in MMR genes. Figure S3. Genomic landscape according to the TMB and MSI status in (A) endometrial, (B) cervical, and (C) ovarian cancer. Each cancer was categorized as TMB-H with MSS, MSI-H (regardless of TMB status), and TMB-L with MSS. Table S1. Distribution of histological subtypes in each cancer. Table S2. Frequency of MSI-H and TMB-H according to the histological subtypes in each cancer. Table S3. Genomic alterations of MMR genes in each cancer with MSI-H.

## References

[1] Naito Y., Aburatani H., Amano T., Baba E., Furukawa T., Hayashida T., Hiyama E., Ikeda S., Kanai M., Kato M. (2021). Clinical practice guidance for next-generation sequencing in cancer diagnosis and treatment (edition 2.1). Int. J. Clin. Oncol..

[2] Yoshii Y., Okazaki S., Takeda M. (2021). Current status of next-generation sequencing-based cancer genome profiling tests in Japan and prospects for liquid biopsy. Life.

[3] Kohno T., Kato M., Kohsaka S., Sudo T., Tamai I., Shiraishi Y., Okuma Y., Ogasawara D., Suzuki T., Yoshida T. (2022). C-CAT: The National Datacenter for Cancer Genomic Medicine in Japan. Cancer Discov..

[4] Levine D. (2013). The Cancer Genome Atlas Research Network. Integrated genomic characterization of endometrial carcinoma. Nature.

[5] Baiden-Amissah R.E.M., Annibali D., Tuyaerts S., Amant F. (2021). Endometrial cancer molecular characterization: The key to identifying high-risk patients and defining guidelines for clinical decision-making?. Cancers.

[6] León-Castillo A., Britton H., McConechy M.K., McAlpine J.N., Nout R., Kommoss S., Brucker S.Y., Carlson J.W., Epstein E., Rau T.T. (2020). Interpretation of somatic POLE mutations in endometrial carcinoma. J. Pathol..

[7] Abu-Rustum N., Yashar C., Arend R., Barber E., Bradley K., Brooks R., Campos S.M., Chino J., Chon H.S., Chu C. (2023). Uterine Neoplasms, Version 1.2023, NCCN Clinical Practice Guidelines in Oncology. J. Natl. Compr. Cancer Netw..

[8] The Cancer Genome Atlas Research Network (2017). Integrated genomic and molecular characterization of cervical cancer. Nature.

[9] Cancer Genome Atlas Research Network (2011). Integrated genomic analyses of ovarian carcinoma. Nature.

[10] Yoshihara K., Baba T., Tokunaga H., Nishino K., Sekine M., Takamatsu S., Matsumura N., Yoshida H., Kajiyama H., Shimada M. (2023). Homologous recombination inquiry through ovarian malignancy investigations: JGOG3025 Study. Cancer Sci..

[11] Wiegand K.C., Shah S.P., Al-Agha O.M., Zhao Y., Tse K., Zeng T., Senz J., McConechy M.K., Anglesio M.S., Kalloger S.E. (2010). ARID1A mutations in endometriosis-associated ovarian carcinomas. N. Engl. J. Med..

[12] Ryland G.L., Hunter S.M., Doyle M.A., Caramia F., Li J., Rowley S.M., Christie M., Allan P.E., Stephens A.N., Bowtell D.D. (2015). Mutational landscape of mucinous ovarian carcinoma and its neoplastic precursors. Genome Med..

[13] Business Wire (2019). Merck’s KEYTRUDA® (Pembrolizumab) Receives Five New Approvals in Japan, Including in Advanced Non–Small Cell Lung Cancer (NSCLC), as Adjuvant Therapy for Melanoma, and in Advanced Microsatellite Instability-High (MSI-H) Tumors.

[14] Merck FDA Approves KEYTRUDA® (Pembrolizumab) plus LENVIMA® (Lenvatinib) Combination for First-Line Treatment of Adult Patients with Advanced Renal Cell Carcinoma (RCC). https://www.merck.com/news/fda-approves-keytruda-pembrolizumab-plus-lenvima-lenvatinib-combination-for-first-line-treatment-of-adult-patients-with-advanced-renal-cell-carcinoma-rcc/.

[15] Tewari K.S., Monk B.J., Vergote I., Miller A., de Melo A.C., Kim H.S., Kim Y.M., Lisyanskaya A., Samouëlian V., Lorusso D. (2022). Survival with cemiplimab in recurrent cervical cancer. N. Engl. J. Med..

[16] Makker V., Colombo N., Casado Herráez A., Santin A.D., Colomba E., Miller D.S., Fujiwara K., Pignata S., Baron-Hay S., Ray-Coquard I. (2022). Lenvatinib plus pembrolizumab for advanced endometrial cancer. N. Engl. J. Med..

[17] Eskander R.N., Sill M.W., Beffa L., Moore R.G., Hope J.M., Musa F.B., Mannel R., Shahin M.S., Cantuaria G.H., Girda E. (2023). Pembrolizumab plus chemotherapy in advanced endometrial cancer. N. Engl. J. Med..

[18] Mirza M.R., Chase D.M., Slomovitz B.M., dePont Christensen R., Novák Z., Black D., Gilbert L., Sharma S., Valabrega G., Landrum L.M. (2023). Dostarlimab for primary advanced or recurrent endometrial cancer. N. Engl. J. Med..

[19] Colombo N., Dubot C., Lorusso D., Caceres M.V., Hasegawa K., Shapira-Frommer R., Tewari K.S., Salman P., Hoyos Usta E., Yañez E. (2021). Pembrolizumab for persistent, recurrent, or metastatic cervical cancer. N. Engl. J. Med..

[20] Colombo I., Karakasis K., Suku S., Oza A.M. (2023). Chasing immune checkpoint inhibitors in ovarian cancer: Novel combinations and biomarker discovery. Cancers.

[21] Milbury C.A., Creeden J., Yip W.K., Smith D.L., Pattani V., Maxwell K., Sawchyn B., Gjoerup O., Meng W., Skoletsky J. (2022). Clinical and analytical validation of FoundationOne^®^CDx, a comprehensive genomic profiling assay for solid tumors. PLoS ONE.

[22] Belli C., Repetto M., Anand S., Porta C., Subbiah V., Curigliano G. (2023). The emerging role of PI3K inhibitors for solid tumour treatment and beyond. Br. J. Cancer.

[23] Bitler B.G., Aird K.M., Garipov A., Li H., Amatangelo M., Kossenkov A.V., Schultz D.C., Liu Q., Shih I.e.M., Conejo-Garcia J.R. (2015). Synthetic lethality by targeting EZH2 methyltransferase activity in ARID1A-mutated cancers. Nat. Med..

[24] Ogiwara H., Takahashi K., Sasaki M., Kuroda T., Yoshida H., Watanabe R., Maruyama A., Makinoshima H., Chiwaki F., Sasaki H. (2019). Targeting the vulnerability of glutathione metabolism in ARID1A-deficient cancers. Cancer Cell.

[25] Rathod L.S., Dabhade P.S., Mokale S.N. (2023). Recent progress in targeting KRAS mutant cancers with covalent G12C-specific inhibitors. Drug Discov. Today.

[26] Escher T.E., Satchell K.J.F. (2023). RAS degraders: The new frontier for RAS-driven cancers. Mol. Ther..

[27] Yamada K., Hori Y., Inoue S., Yamamoto Y., Iso K., Kamiyama H., Yamaguchi A., Kimura T., Uesugi M., Ito J. (2021). E7386, a selective inhibitor of the interaction between β-catenin and CBP, exerts antitumor activity in tumor models with activated canonical Wnt signaling. Cancer Res..

[28] Nishikawa T., Hasegawa K., Matsumoto K., Mori M., Hirashima Y., Takehara K., Ariyoshi K., Kato T., Yagishita S., Hamada A. (2023). Trastuzumab deruxtecan for human epidermal growth factor receptor 2-expressing advanced or recurrent uterine carcinosarcoma (NCCH1615): The STATICE trial. J. Clin. Oncol..

[29] Shigeta S., Shimada M., Suzuki S., Kajiyama H., Oda K., Takehara K., Mandai M., Aoki D., Enomoto T., Okamoto A. (2023). An attempt to develop a new treatment strategy for rare refractory gynecological malignancies: The Japanese Gynecologic Oncology Group. JMA J..

[30] Oda K., Aoki D., Tsuda H., Nishihara H., Aoyama H., Inomata H., Shimada M., Enomoto T. (2023). Japanese nationwide observational multicenter study of tumor BRCA1/2 variant testing in advanced ovarian cancer. Cancer Sci..

[31] Kage H., Oda K., Muto M., Tsuchihara K., Okita N., Okuma Y., Kikuchi J., Shirota H., Hayashi H., Kokuryo T. (2023). Human resources for administrative work to carry out a comprehensive genomic profiling test in Japan. Cancer Sci..

[32] Talhouk A., McConechy M.K., Leung S., Yang W., Lum A., Senz J., Boyd N., Pike J., Anglesio M., Kwon J.S. (2017). Confirmation of ProMisE: A simple, genomics-based clinical classifier for endometrial cancer. Cancer.

[33] Liu J.F., Xiong N., Campos S.M., Wright A.A., Krasner C., Schumer S., Horowitz N., Veneris J., Tayob N., Morrissey S. (2021). Phase II study of the WEE1 inhibitor adavosertib in recurrent uterine serous carcinoma. J. Clin. Oncol..

[34] Liu J., Oza A.M., Colombo N., Oaknin A. (2022). ADAGIO: A phase IIb international study of the Wee1 inhibitor adavosertib in women with recurrent or persistent uterine serous carcinoma. Int. J. Gynecol. Cancer.

[35] Stolnicu S. (2021). Cervical cancer: What’s new in classification, morphology, molecular findings and prognosis of glandular precursor and invasive lesions. Diagn. Histopathol..

[36] Pal A., Kundu R. (2020). Human papillomavirus E6 and E7: The cervical cancer hallmarks and targets for therapy. Front. Microbiol..

[37] Nishio S., Mikami Y., Tokunaga H., Yaegashi N., Satoh T., Saito M., Okamoto A., Kasamatsu T., Miyamoto T., Shiozawa T. (2019). Analysis of gastric-type mucinous carcinoma of the uterine cervix—An aggressive tumor with a poor prognosis: A multi-institutional study. Gynecol. Oncol..

[38] Asano H., Oda K., Yoshihara K., Ito Y.M., Matsumura N., Shimada M., Watari H., Enomoto T. (2022). Phase II study of niraparib in recurrent or persistent rare fraction of gynecologic malignancies with homologous recombination deficiency (JGOG2052). J. Gynecol. Oncol..

[39] Akagi G., Oki E., Taniguchi H., Nakatani K., Aoki D., Kuwata T., Yoshino T. (2021). The real-world data on microsatellite instability status in various unresectable or metastatic solid tumors. Cancer Sci..

[40] Mishima S., Naito Y., Akagi K., Hayashi N., Hirasawa A., Hishiki T., Igarashi A., Ikeda M., Kadowaki S., Kajiyama H. (2023). Japanese Society of Medical Oncology/Japan Society of Clinical Oncology/Japanese Society of Pediatric Hematology/Oncology-led clinical recommendations on the diagnosis and use of immunotherapy in patients with high tumor mutational burden tumors. Int. J. Clin. Oncol..

[41] Xu Y., Zuo F., Wang H., Jing J., He X. (2022). The current landscape of predictive and prognostic biomarkers for immune checkpoint blockade in ovarian cancer. Front. Immunol..

