# Supplementary material for: Genomic Landscape of Endometrial, Ovarian, and Cervical Cancers in Japan from the Database in the Center for Cancer Genomics and Advanced Therapeutics
Source: Cancers (Basel). 2023 Dec 27;16(1):136. doi: 10.3390/cancers16010136 (PMC10778092; doi:10.3390/cancers16010136)
Supplement: Supplementary file 1 [file cancers-16-00136-s001.zip › Figure S1. Genomic landscape of three gynecological cancers.pdf]

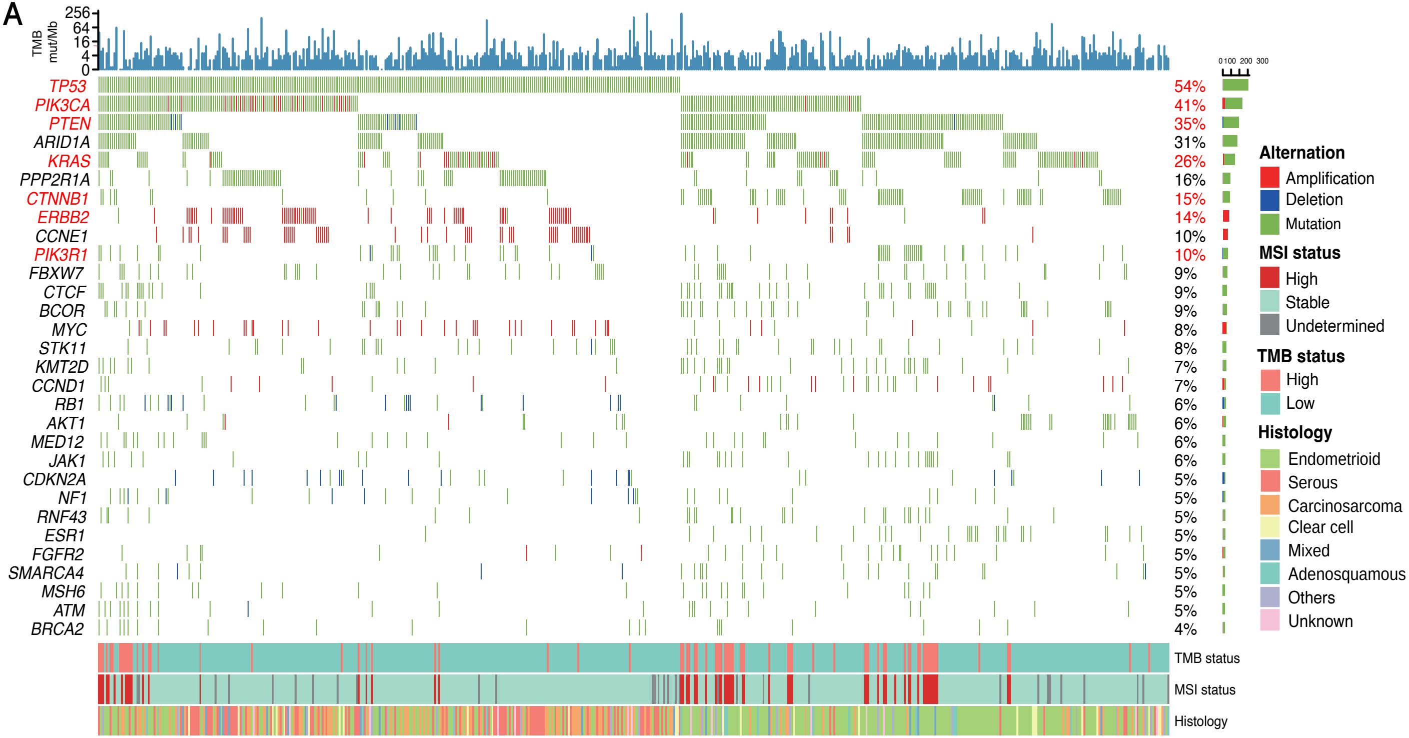
A
TMB mut/Mb
256
64
16
4
0
TP53 54%
PIK3CA 41%
PTEN 35%
ARID1A 31%
KRAS 26%
PPP2R1A 16%
CTNNB1 15%
ERBB2 14%
CCNE1 10%
PIK3R1 10%
FBXW7 9%
CTCF 9%
BCOR 9%
MYC 8%
STK11 8%
KMT2D 7%
CCND1 7%
RB1 6%
AKT1 6%
MED12 6%
JAK1 6%
CDKN2A 5%
NF1 5%
RNF43 5%
ESR1 5%
FGFR2 5%
SMARCA4 5%
MSH6 5%
ATM 5%
BRCA2 4%
0 100 200 300
Alternation
Amplification
Deletion
Mutation
MSI status
High
Stable
Undetermined
TMB status
High
Low
Histology
Endometrioid
Serous
Carcinosarcoma
Clear cell
Mixed
Adenosquamous
Others
Unknown
TMB status
MSI status
Histology

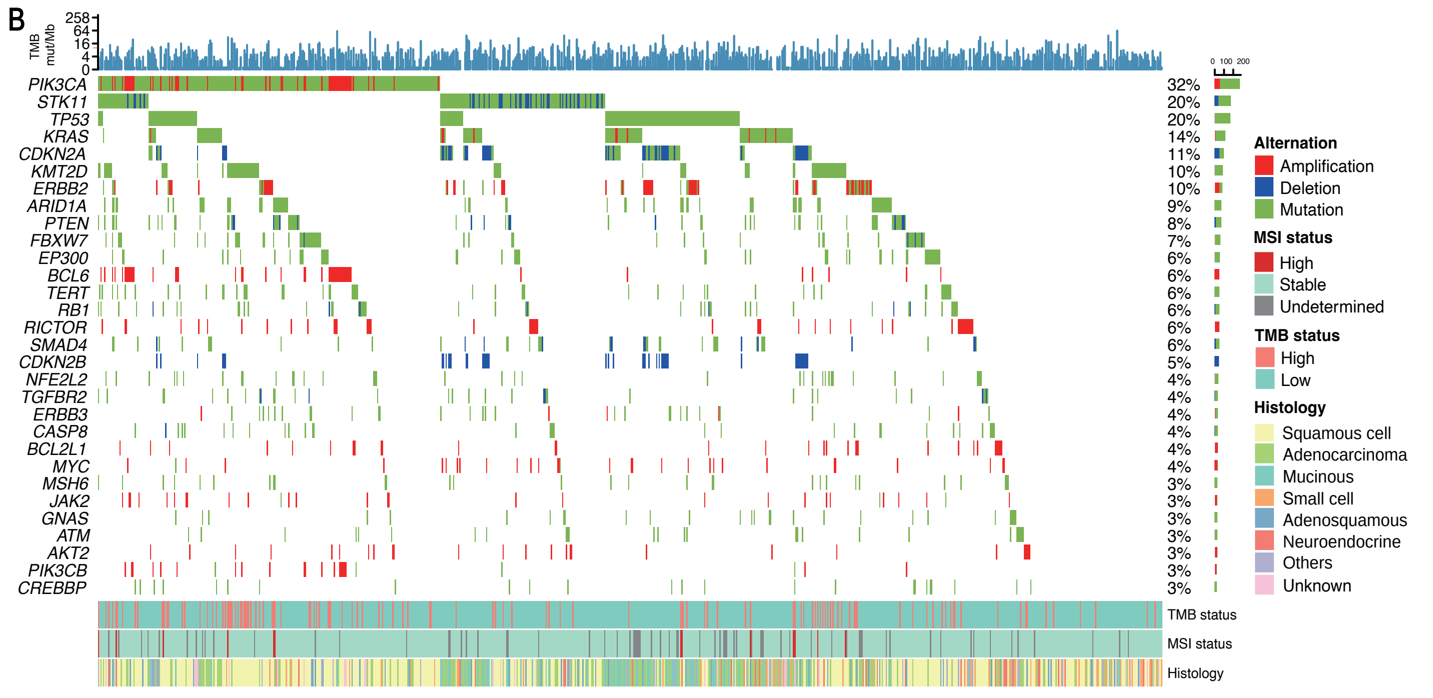
B
TMB mut/Mb
258
64
16
4
0
PIK3CA 32%
STK11 20%
TP53 20%
KRAS 14%
CDKN2A 11%
KMT2D 10%
ERBB2 10%
ARID1A 9%
PTEN 8%
FBXW7 7%
EP300 6%
BCL6 6%
TERT 6%
RB1 6%
RICTOR 6%
SMAD4 6%
CDKN2B 5%
NFE2L2 4%
TGFBR2 4%
ERBB3 4%
CASP8 4%
BCL2L1 4%
MYC 4%
MSH6 3%
JAK2 3%
GNAS 3%
ATM 3%
AKT2 3%
PIK3CB 3%
CREBBP 3%
0 100 200
TMB status
MSI status
Histology
Alternation
Amplification
Deletion
Mutation
MSI status
High
Stable
Undetermined
TMB status
High
Low
Histology
Squamous cell
Adenocarcinoma
Mucinous
Small cell
Adenosquamous
Neuroendocrine
Others
Unknown

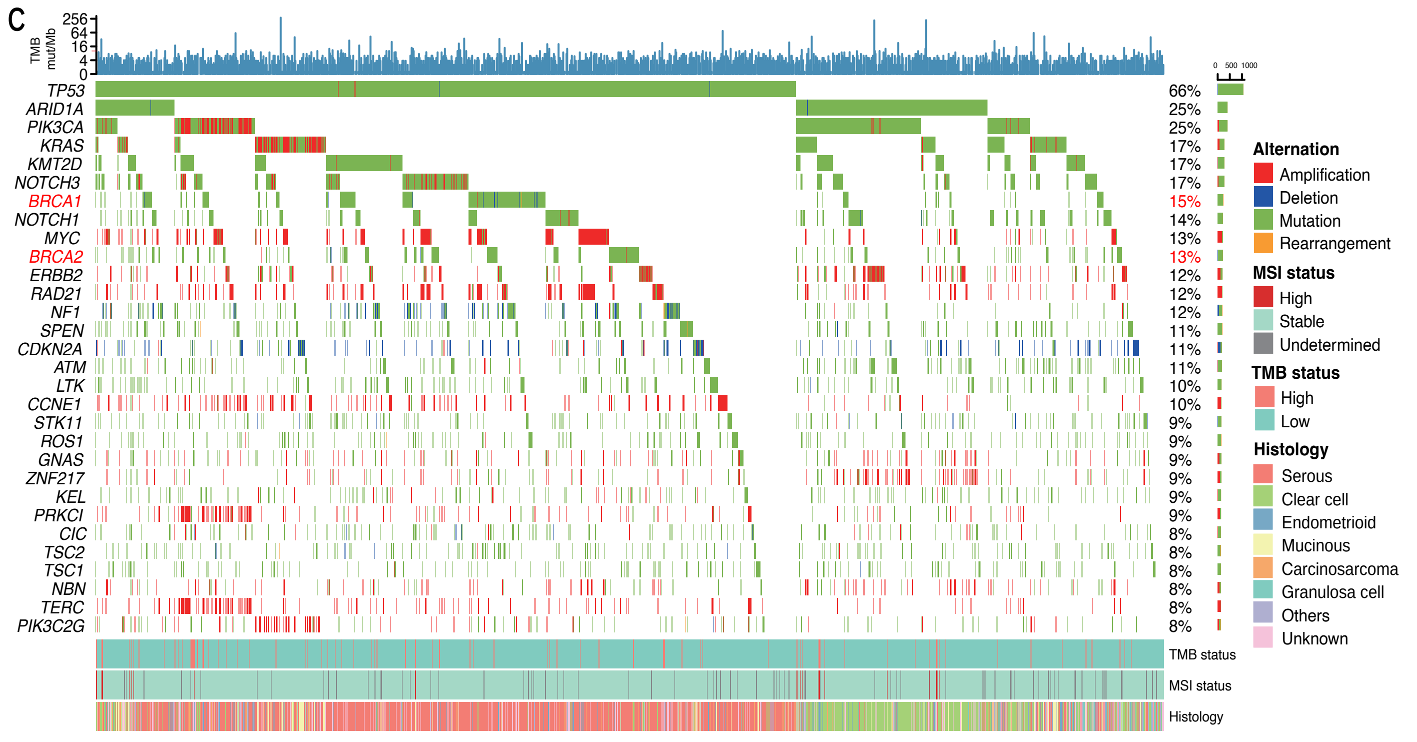
C
TMB mut/Mb
256
64
16
4
0
0 500 1000
TP53 66%
ARID1A 25%
PIK3CA 25%
KRAS 17%
KMT2D 17%
NOTCH3 17%
BRCA1 15%
NOTCH1 14%
MYC 13%
BRCA2 13%
ERBB2 12%
RAD21 12%
NF1 12%
SPEN 11%
CDKN2A 11%
ATM 11%
LTK 10%
CCNE1 10%
STK11 9%
ROS1 9%
GNAS 9%
ZNF217 9%
KEL 9%
PRKCI 9%
CIC 8%
TSC2 8%
TSC1 8%
NBN 8%
TERC 8%
PIK3C2G 8%
TMB status
MSI status
Histology
Alternation
Amplification
Deletion
Mutation
Rearrangement
MSI status
High
Stable
Undetermined
TMB status
High
Low
Histology
Serous
Clear cell
Endometrioid
Mucinous
Carcinosarcoma
Granulosa cell
Others
Unknown

Supplementary Figure S1. Genomic landscape of three gynecological cancers. Recurrently mutated genes are listed with the status of TMB and MSI and with information about types of alterations and histological subtypes in (A) endometrial, (B) cervical, and (C) ovarian cancers. The upper plot represents the TMB scores by F1CDx.Waterfall plot of genetic alteration profiles in endometrial (A), cervical (B), and ovarian cancer (C).
