# Supplementary material for: Genomic Landscape of Endometrial, Ovarian, and Cervical Cancers in Japan from the Database in the Center for Cancer Genomics and Advanced Therapeutics
Source: Cancers (Basel). 2023 Dec 27;16(1):136. doi: 10.3390/cancers16010136 (PMC10778092; doi:10.3390/cancers16010136)
Supplement: Supplementary file 1 [file cancers-16-00136-s001.zip › Figure S2. Frequency of genomic alterations in the MMR genes according to the MSI and TMB stauts.pdf]

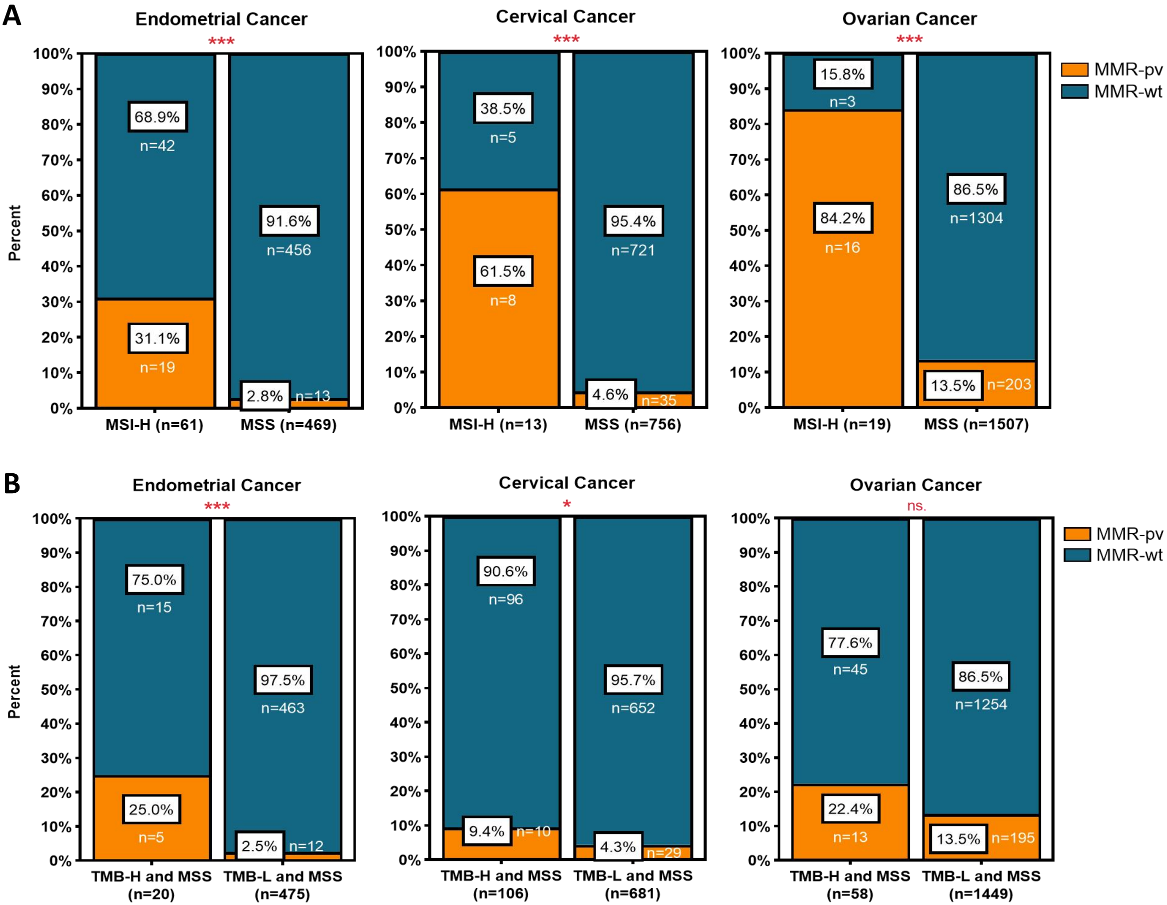
A
Endometrial Cancer
***
Cervical Cancer
***
Ovarian Cancer
***
MMR-pv
MMR-wt
Percent
100%
90%
80%
70%
60%
50%
40%
30%
20%
10%
0%
68.9%
n=42
31.1%
n=19
91.6%
n=456
2.8%
n=13
38.5%
n=5
61.5%
n=8
95.4%
n=721
4.6%
n=35
15.8%
n=3
84.2%
n=16
86.5%
n=1304
13.5%
n=203
MSI-H (n=61)
MSS (n=469)
MSI-H (n=13)
MSS (n=756)
MSI-H (n=19)
MSS (n=1507)
B
Endometrial Cancer
***
Cervical Cancer
*
Ovarian Cancer
ns.
MMR-pv
MMR-wt
Percent
75.0%
n=15
25.0%
n=5
97.5%
n=463
2.5%
n=12
90.6%
n=96
9.4%
n=10
95.7%
n=652
4.3%
n=29
77.6%
n=45
22.4%
n=13
86.5%
n=1254
13.5%
n=195
TMB-H and MSS (n=20)
TMB-L and MSS (n=475)
TMB-H and MSS (n=106)
TMB-L and MSS (n=681)
TMB-H and MSS (n=58)
TMB-L and MSS (n=1449)

Supplementary Figure S2. Frequency of genomic alterations in the mismatch repair (MMR) genes according to the MSI and TMB stauts in each cancer type. (A) Frequency of MMR alterations according to the MSI status, (B) Frequency of MMR alterations according to the TMB status. Comparisons between the groups were performed by Fisher's exact test (* P<0.05; ** P<0.01; *** P<0.001). MMR-pv, pathogenic variants in MMR genes, MMR-wt, no pathogenic variants (wild-type) in MMR genes.
