# Supplementary material for: Genomic Landscape of Endometrial, Ovarian, and Cervical Cancers in Japan from the Database in the Center for Cancer Genomics and Advanced Therapeutics
Source: Cancers (Basel). 2023 Dec 27;16(1):136. doi: 10.3390/cancers16010136 (PMC10778092; doi:10.3390/cancers16010136)
Supplement: Supplementary file 1 [file cancers-16-00136-s001.zip › Figure S3. Genomic landscape according to the TMB and MSI status in endometrial, cervical, and ovarian cancer.pdf]

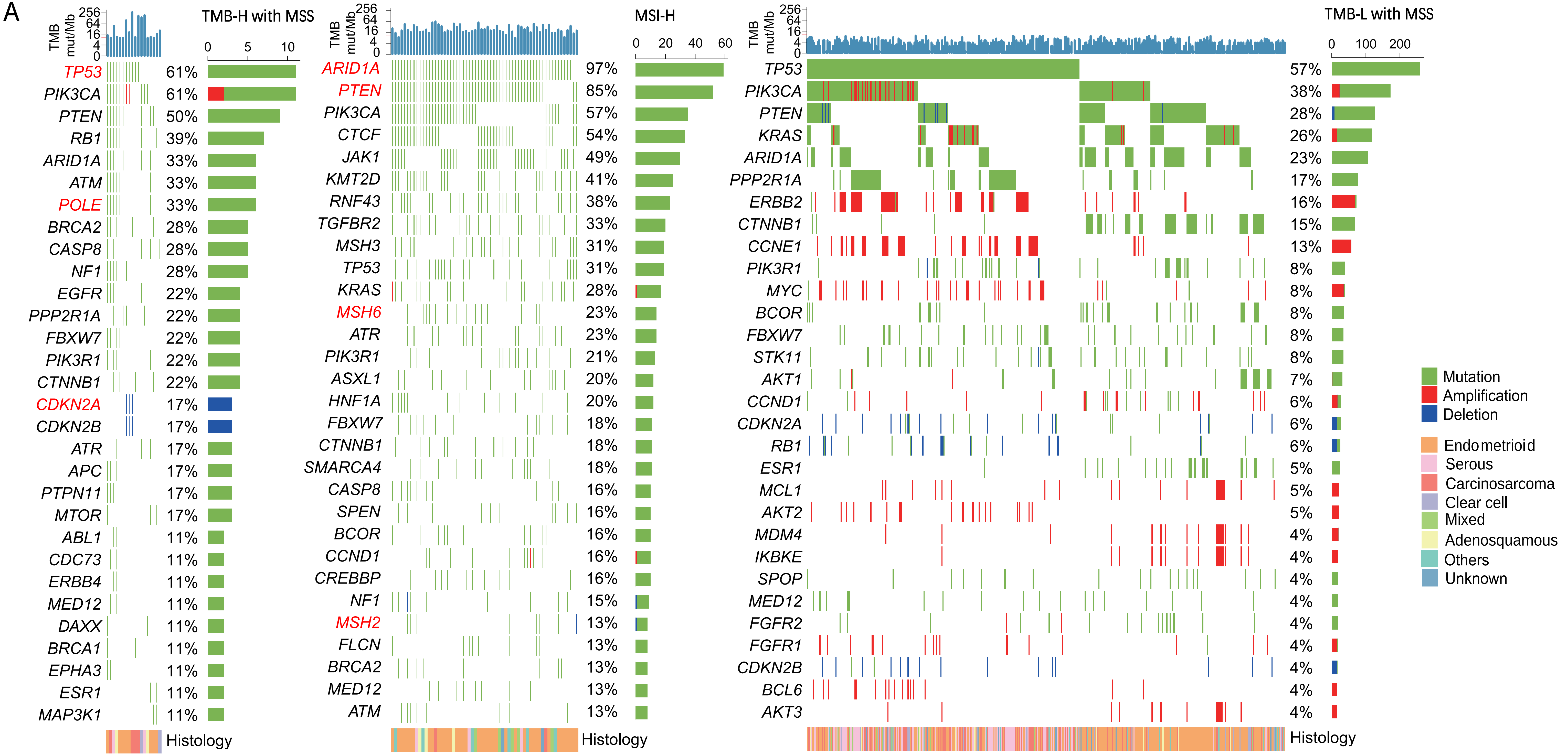

A
TMB-H with MSS
MSI-H
TMB-L with MSS
TMB mut/Mb
TP53 61%
PIK3CA 61%
PTEN 50%
RB1 39%
ARID1A 33%
ATM 33%
POLE 33%
BRCA2 28%
CASP8 28%
NF1 28%
EGFR 22%
PPP2R1A 22%
FBXW7 22%
PIK3R1 22%
CTNNB1 22%
CDKN2A 17%
CDKN2B 17%
ATR 17%
APC 17%
PTPN11 17%
MTOR 17%
ABL1 11%
CDC73 11%
ERBB4 11%
MED12 11%
DAXX 11%
BRCA1 11%
EPHA3 11%
ESR1 11%
MAP3K1 11%
ARID1A 97%
PTEN 85%
PIK3CA 57%
CTCF 54%
JAK1 49%
KMT2D 41%
RNF43 38%
TGFBR2 33%
MSH3 31%
TP53 31%
KRAS 28%
MSH6 23%
ATR 23%
PIK3R1 21%
ASXL1 20%
HNF1A 20%
FBXW7 18%
CTNNB1 18%
SMARCA4 18%
CASP8 16%
SPEN 16%
BCOR 16%
CCND1 16%
CREBBP 16%
NF1 15%
MSH2 13%
FLCN 13%
BRCA2 13%
MED12 13%
ATM 13%
TP53 57%
PIK3CA 38%
PTEN 28%
KRAS 26%
ARID1A 23%
PPP2R1A 17%
ERBB2 16%
CTNNB1 15%
CCNE1 13%
PIK3R1 8%
MYC 8%
BCOR 8%
FBXW7 8%
STK11 8%
AKT1 7%
CCND1 6%
CDKN2A 6%
RB1 6%
ESR1 5%
MCL1 5%
AKT2 5%
MDM4 4%
IKBKE 4%
SPOP 4%
MED12 4%
FGFR2 4%
FGFR1 4%
CDKN2B 4%
BCL6 4%
AKT3 4%
Histology
Mutation
Amplification
Deletion
Endometrioid
Serous
Carcinosarcoma
Clear cell
Mixed
Adenosquamous
Others
Unknown

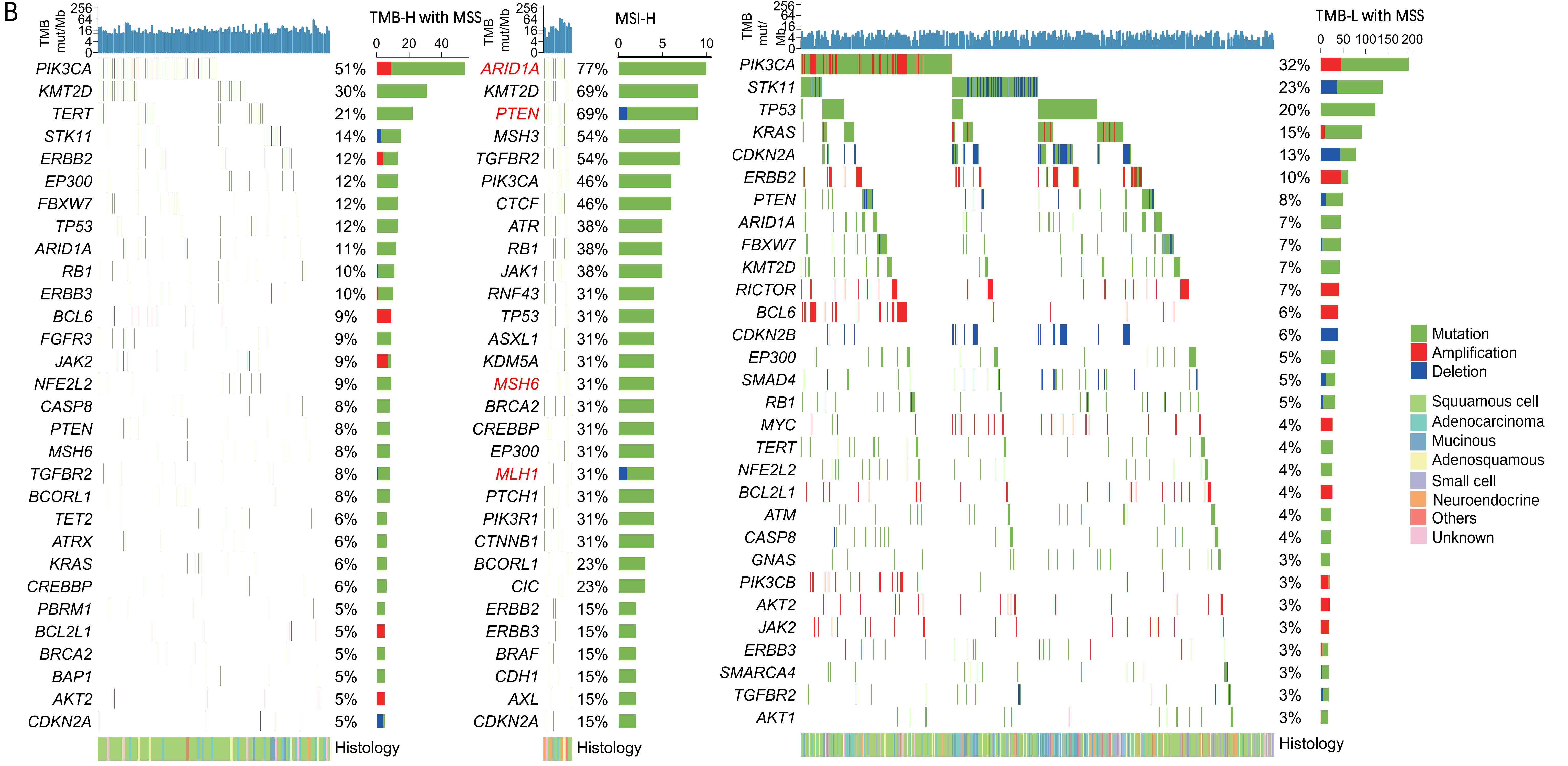
B
TMB mut/Mb
256
64
16
4
0
TMB-H with MSS
0 20 40
PIK3CA 51%
KMT2D 30%
TERT 21%
STK11 14%
ERBB2 12%
EP300 12%
FBXW7 12%
TP53 12%
ARID1A 11%
RB1 10%
ERBB3 10%
BCL6 9%
FGFR3 9%
JAK2 9%
NFE2L2 9%
CASP8 8%
PTEN 8%
MSH6 8%
TGFBR2 8%
BCORL1 8%
TET2 6%
ATRX 6%
KRAS 6%
CREBBP 6%
PBRM1 5%
BCL2L1 5%
BRCA2 5%
BAP1 5%
AKT2 5%
CDKN2A 5%
Histology
MSI-H
0 5 10
ARID1A 77%
KMT2D 69%
PTEN 69%
MSH3 54%
TGFBR2 54%
PIK3CA 46%
CTCF 46%
ATR 38%
RB1 38%
JAK1 38%
RNF43 31%
TP53 31%
ASXL1 31%
KDM5A 31%
MSH6 31%
BRCA2 31%
CREBBP 31%
EP300 31%
MLH1 31%
PTCH1 31%
PIK3R1 31%
CTNNB1 31%
BCORL1 23%
CIC 23%
ERBB2 15%
ERBB3 15%
BRAF 15%
CDH1 15%
AXL 15%
CDKN2A 15%
Histology
TMB-L with MSS
0 50 100 150 200
PIK3CA 32%
STK11 23%
TP53 20%
KRAS 15%
CDKN2A 13%
ERBB2 10%
PTEN 8%
ARID1A 7%
FBXW7 7%
KMT2D 7%
RICTOR 7%
BCL6 6%
CDKN2B 6%
EP300 5%
SMAD4 5%
RB1 5%
MYC 4%
TERT 4%
NFE2L2 4%
BCL2L1 4%
ATM 4%
CASP8 4%
GNAS 3%
PIK3CB 3%
AKT2 3%
JAK2 3%
ERBB3 3%
SMARCA4 3%
TGFBR2 3%
AKT1 3%
Histology
Mutation
Amplification
Deletion
Squuamous cell
Adenocarcinoma
Mucinous
Adenosquamous
Small cell
Neuroendocrine
Others
Unknown

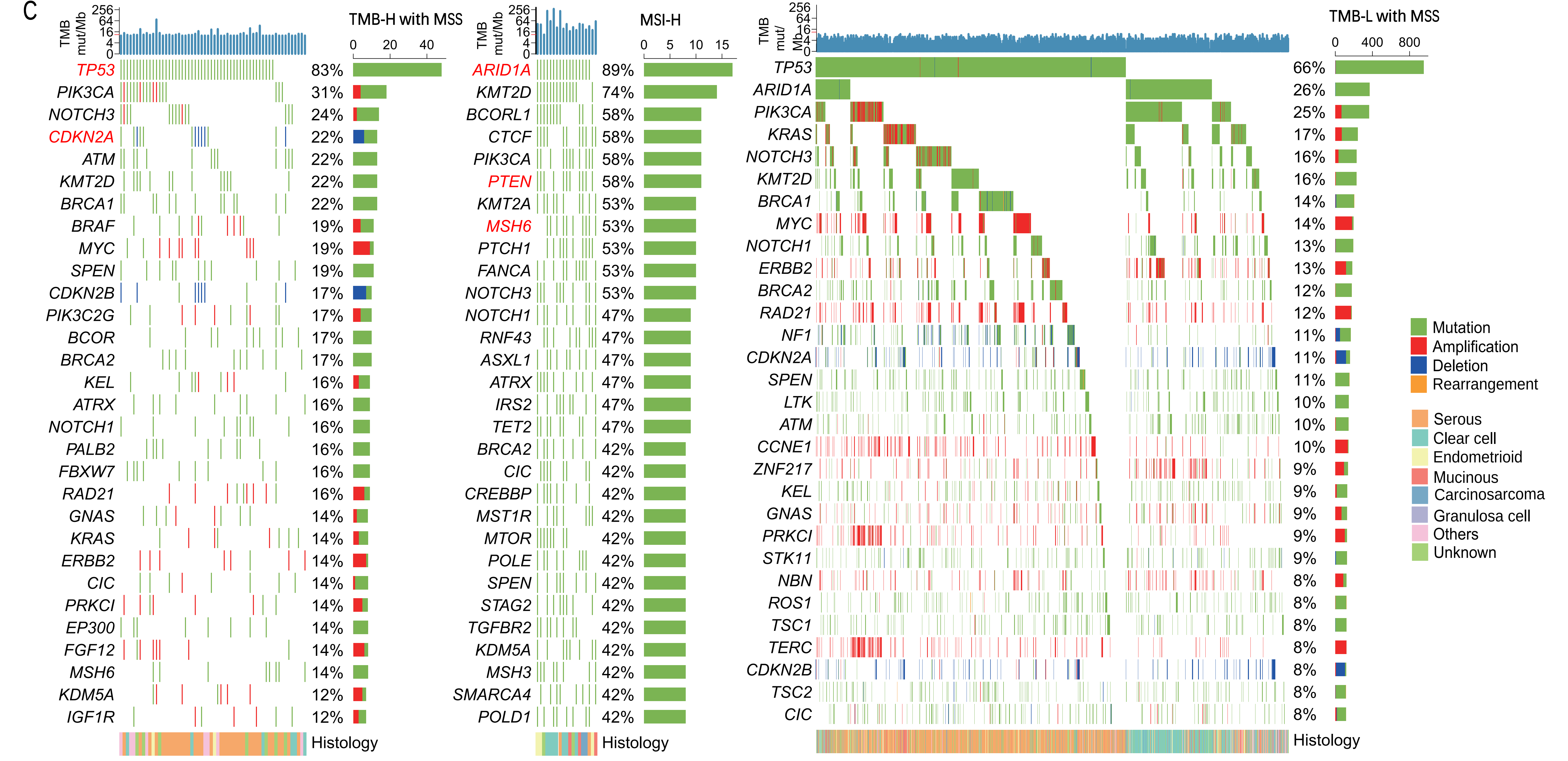
C
TMB mut/Mb
256
64
16
4
0
TMB-H with MSS
0
20
40
TP53 83%
PIK3CA 31%
NOTCH3 24%
CDKN2A 22%
ATM 22%
KMT2D 22%
BRCA1 22%
BRAF 19%
MYC 19%
SPEN 19%
CDKN2B 17%
PIK3C2G 17%
BCOR 17%
BRCA2 17%
KEL 16%
ATRX 16%
NOTCH1 16%
PALB2 16%
FBXW7 16%
RAD21 16%
GNAS 14%
KRAS 14%
ERBB2 14%
CIC 14%
PRKCI 14%
EP300 14%
FGF12 14%
MSH6 14%
KDM5A 12%
IGF1R 12%
Histology
MSI-H
0
5
10
15
ARID1A 89%
KMT2D 74%
BCORL1 58%
CTCF 58%
PIK3CA 58%
PTEN 58%
KMT2A 53%
MSH6 53%
PTCH1 53%
FANCA 53%
NOTCH3 53%
NOTCH1 47%
RNF43 47%
ASXL1 47%
ATRX 47%
IRS2 47%
TET2 47%
BRCA2 42%
CIC 42%
CREBBP 42%
MST1R 42%
MTOR 42%
POLE 42%
SPEN 42%
STAG2 42%
TGFBR2 42%
KDM5A 42%
MSH3 42%
SMARCA4 42%
POLD1 42%
Histology
TMB-L with MSS
0
400
800
TP53 66%
ARID1A 26%
PIK3CA 25%
KRAS 17%
NOTCH3 16%
KMT2D 16%
BRCA1 14%
MYC 14%
NOTCH1 13%
ERBB2 13%
BRCA2 12%
RAD21 12%
NF1 11%
CDKN2A 11%
SPEN 11%
LTK 10%
ATM 10%
CCNE1 10%
ZNF217 9%
KEL 9%
GNAS 9%
PRKCI 9%
STK11 9%
NBN 8%
ROS1 8%
TSC1 8%
TERC 8%
CDKN2B 8%
TSC2 8%
CIC 8%
Histology
Mutation
Amplification
Deletion
Rearrangement
Serous
Clear cell
Endometrioid
Mucinous
Carcinosarcoma
Granulosa cell
Others
Unknown

Supplementary Figure S3. Genomic landscape according to the TMB and MSI status in (A) endometrial, (B) cervical, and (C) ovarian cancer. Each cancer was categorized as TMB-H with MSS, MSI-H (regardless of TMB status), and TMB-L with MSS.
