# Supplementary material for: Genomic Landscape of Endometrial, Ovarian, and Cervical Cancers in Japan from the Database in the Center for Cancer Genomics and Advanced Therapeutics
Source: Cancers (Basel). 2023 Dec 27;16(1):136. doi: 10.3390/cancers16010136 (PMC10778092; doi:10.3390/cancers16010136)
Supplement: Supplementary file 1 [file cancers-16-00136-s001.zip › Table S1. Distribution of histological subtypes in each cancer.pdf]

Table S1. Distribution of histological subtypes in each cancer

**Endometrial Cancer**		
Endometrioid carcinoma	275	49.0%
Serous carcinoma	102	18.2%
Carcinosarcoma	87	15.5%
Clear cell carcinoma	26	4.6%
Mixed carcinoma	18	3.2%
Adenosquamous carcinoma	17	3.0%
Dedifferentiated carcinoma	10	1.8%
Undifferentiated carcinoma	5	0.89%
Neuroendocrine carcinoma	4	0.71%
Poorly differentiated carcinoma	3	0.53%
Unknown	14	2.5%
Total	561	

**Cervical Cancer**		
Total		839
Squamous cell carcinoma	389	46.4%
Adenocarcinoma	180	21.5%
Mucinous carcinoma	80	9.5%
Small cell carcinoma	48	5.7%
Adenosquamous carcinoma	46	5.5%
Neuroendocrine carcinoma	35	4.2%
Mixed carcinoma	8	0.95%
Endometrioid carcinoma	7	0.83%
Clear cell carcinoma	6	0.72%
Serous carcinoma	3	0.36%
Carcinosarcoma	3	0.36%
Adenoid cystic carcinoma	1	0.12%
Glassy cell carcinoma	1	0.12%
Sarcoma	2	0.24%
Unknown	30	3.6%
Total	839	

**Ovarian Cancer**		
Serous carcinoma	784	48.8%
Clear cell carcinoma	333	20.7%
Endometrioid carcinoma	92	5.7%
Mucinous carcinoma	91	5.7%
Carcinosarcoma	55	3.4%
Seromucinous carcinoma	8	0.50%
Serous borderline ovarian tumor	5	0.31%
Small cell carcinoma	5	0.31%
Brenner tumor	1	0.06%
Granulosa cell tumor	26	1.6%
Mature/Immature teratoma	23	1.4%
Germ cell tumor	5	0.31%
Yolk sac tumor	4	0.25%
Sertoli-Leydig cell tumor	2	0.12%
Dysgerminoma	1	0.06%
Mixed germ cell tumor	1	0.06%
Steroid cell tumor	1	0.06%
Unknown	169	10.5%
Total	1,606	

*Non-epithelial tumors are marked in color
