# Supplementary material for: Genomic Landscape of Endometrial, Ovarian, and Cervical Cancers in Japan from the Database in the Center for Cancer Genomics and Advanced Therapeutics
Source: Cancers (Basel). 2023 Dec 27;16(1):136. doi: 10.3390/cancers16010136 (PMC10778092; doi:10.3390/cancers16010136)
Supplement: Supplementary file 1 [file cancers-16-00136-s001.zip › Table S2. Frequency of MSI-H and TMB-H according to the histological subtypes in each cancer.pdf]

Table S2. Frequency of MSI-H and TMB-H according to the histological subtypes in each cancer.

**Endometrial Cancer**	Total		MSI-H		TMB-H	
	No. of Patients (%)		No. of Patients (% of respective subtypes)			
Total	561		61	10.9%	78	13.9%
Endometrioid endometrial carcinoma	275	49.0%	40	14.5%	47	17.1%
Endometrial serous carcinoma	102	18.2%	3	2.9%	5	4.9%
Uterine carcinosarcoma	87	15.5%	2	2.3%	6	6.9%
Uterine clear cell carcinoma	26	4.6%	0	0.0%	2	7.7%
Mixed endometrial carcinoma	18	3.2%	5	27.8%	5	27.8%
Uterine adenosquamous carcinoma	17	3.0%	3	17.6%	5	29.4%
Others	22	3.9%	6	27.3%	6	27.3%
Unknown	14	2.5%	2	14.3%	2	14.3%

**Cervical Cancer**	Total		MSI-H		TMB-H	
	No. of Patients (%)		No. of Patients (% of respective subtypes)			
Total	839		13	1.5%	119	14.2%
Cervical squamous cell carcinoma	389	46.4%	5	1.3%	80	20.6%
Cervical adenocarcinoma	180	21.5%	2	1.1%	15	8.3%
Cervical mucinous carcinoma	80	9.5%	0	0.0%	4	5.0%
Small cell carcinoma of cervix	48	5.7%	0	0.0%	0	0.0%
Cervical adenosquamous carcinoma	46	5.5%	1	2.2%	6	13.0%
Cervical neuroendocrine carcinoma	35	4.2%	3	8.6%	5	14.3%
Others	31	3.7%	1	3.2%	2	6.5%
Unknown	30	3.6%	1	3.3%	7	23.3%

**Ovarian Cancer**	Total		MSI-H		TMB-H	
	No. of Patients (%)		No. of Patients (% of respective subtypes)			
Total	1606		19	1.2%	80	5.0%
Serous ovarian carcinoma	784	48.8%	2	0.3%	37	4.7%
Clear cell ovarian carcinoma	333	20.7%	7	2.1%	15	4.5%
Endometrioid ovarian cancer	92	5.7%	3	3.3%	6	6.5%
Mucinous ovarian cancer	91	5.7%	3	3.3%	3	3.3%
Ovarian carcinosarcoma	55	3.4%	2	3.6%	2	3.6%
Others	56	3.5%	0	0.0%	8	14.3%
Unknown	169	10.5%	2	1.2%	9	5.3%
