# Supplementary material for: Genomic Landscape of Endometrial, Ovarian, and Cervical Cancers in Japan from the Database in the Center for Cancer Genomics and Advanced Therapeutics
Source: Cancers (Basel). 2023 Dec 27;16(1):136. doi: 10.3390/cancers16010136 (PMC10778092; doi:10.3390/cancers16010136)
Supplement: Supplementary file 1 [file cancers-16-00136-s001.zip › Table S3. Genomic alterations of MMR genes in each cancer with MSI-H.pdf]

Table S3. Genomic alterations of MMR genes in each cancer with MSI-H

	MSH6		*MSH2*		*MLH1*		*PMS2*	
Endometrial Cancer with MSI-H (n=61)	14	23.0%	8	13.1%	4	6.6%	1	1.6%
Cervical Cancer with MSI-H (n=13)	4	30.8%	2	15.4%	4	30.8%	1	7.7%
Ovarian Cancer with MSI-H (n=19)	10	52.6%	7	36.8%	6	31.6%	2	10.5%
